# Synthetic mycolates derivatives to decipher protein mycoloylation, a unique post-translational modification in bacteria

**DOI:** 10.1016/j.jbc.2025.108243

**Published:** 2025-01-27

**Authors:** Emilie Lesur, Yijie Zhang, Nathalie Dautin, Christiane Dietrich, Ines Li de la Sierra-Gallay, Luis A. Augusto, Paulin Rollando, Noureddine Lazar, Dominique Urban, Gilles Doisneau, Florence Constantinesco-Becker, Herman Van Tilbeurgh, Dominique Guianvarc'h, Yann Bourdreux, Nicolas Bayan

**Affiliations:** 1 Université Paris-Saclay, CNRS, Institut de Chimie Moléculaire et des Matériaux d'Orsay (ICMMO), Orsay, France; 2 Université Paris-Saclay, CEA, CNRS, Institute for Integrative Biology of the Cell (I2BC), Gif-sur-Yvette, France

**Keywords:** membrane, lipid metabolism, post-translational modification, mycoloyltransferase, protein lipidation, mycobacteria, mycolyltransferase

## Abstract

Protein mycoloylation is a newly characterized post-translational modification (PTM) specifically found in *Corynebacteriales*, an order of bacteria that includes numerous human pathogens. Their envelope is composed of a unique outer membrane, the so-called mycomembrane made of very-long chain fatty acids, named mycolic acids. Recently, some mycomembrane proteins including PorA have been unambiguously shown to be covalently modified with mycolic acids in the model organism *Corynebacterium glutamicum* by a mechanism that relies on the mycoloyltransferase MytC. This PTM represents the first example of protein *O*-acylation in prokaryotes and the first example of protein modification by mycolic acid. Through the design and synthesis of trehalose monomycolate (TMM) analogs, we prove that *i*) MytC is the mycoloyltransferase directly involved in this PTM, *ii*) TMM, but not trehalose dimycolate (TDM), is a suitable mycolate donor for PorA mycoloylation, *iii*) MytC is able to discriminate between an acyl and a mycoloyl chain *in vitro* unlike other trehalose mycoloyltransferases. We also solved the structure of MytC acyl-enzyme obtained with a soluble short TMM analogs which constitutes the first mycoloyltransferase structure covalently linked to an authentic mycolic acid moiety. These data highlight the great conformational flexibility of the active site of MytC during the reaction cycle and pave the way for a better understanding of the catalytic mechanism of all members of the mycoloyltransferase family including the essential Antigen85 enzymes in *Mycobacteria*.

*Corynebacteriales* are an order of diderm actinomycetes. Their envelope is constituted of an inner membrane and a thick peptidoglycan-arabinogalactan (PG-AG) polymer on which a specific outer membrane is covalently anchored. All members of *Corynebacteriales* produce long α−ramified and β-hydroxylated fatty acids called mycolic acids (1). These unique molecules are produced in the cytoplasm by condensation of two fatty acid molecules that are immediately esterified on trehalose by a polyketide synthase (Pks13) (2, 3). The resulting trehalose monomycolate (TMM) is then translocated across the inner membrane by dedicated Resistance-Nodulation-Division (*RND*) family transporters (4) and serves as a donor of mycolate chain for various envelope acceptors such as TMM itself to form trehalose dimycolate (TDM) or, alternatively, for some arabinosyl terminal ends of arabinogalactan (AG) (1). The resulting mycoloylated external surface of the AG polymer will constitute the outer membrane inner leaflet while TDM together with some other specific lipids will form the outer leaflet of this peculiar membrane, also called the mycomembrane (5). The mycolate transfer from TMM to various acceptors are known to be catalyzed by mycoloyltransferases (Myts), a family of enzymes specifically present in all members of *Corynebacteriales* (6). Several paralogs of Myts coexist in a given species and their exact specificity *in vivo* is still elusive. In *C. glutamicum* 13032, six mycoloyltransferases (MytA-E) were identified. MytA and MytB are the most abundant and have partially redundant functions since they are able to partially replace each other in TMM and arabinose mycoloylation (7, 8).

The mycomembrane is unique to *Corynebacteriales* and reminiscent of the lipopolysaccharide (LPS) outer membrane of Gram-negative bacteria. It represents a hydrophobic barrier protecting the cell from noxious hydrophilic compounds and its permeability to small solutes is strictly determined by porins, which have been described in several genera of *Corynebacteriales*. In fast-growing mycobacteria, MspA is the main porin so far described and its structure is clearly related to classical β-barrels (9) of LPS-containing outer membranes. In slow-growing bacteria, some proteins from the PE/PPE family (which have conserved *N*-terminal domain with a signature proline-glutamate (PE) or proline-proline-glutamate (PPE) motifs) seem to fulfill this function, although their mycomembrane-embedded structure is still unknown (10). Finally, in *Corynebacteria*, small channel-forming proteins have been described but their structure and their function *in vivo* are still very poorly documented (11, 12). In *C. glutamicum* PorAH and PorBC account for a cationic and an anionic porin respectively with estimated conductances of about 2.5 nS and 0.7 nS. Mutants deleted for PorA/PorH and grown on citrate, which blocks PorBC porin, are affected for growth. PorAH is constituted of two small polypeptidic chains of 45 (PorA) and 57 amino acids (PorH), both devoid of any signal sequence (Twin Arginine Translocation (TAT) or general Secretion (Sec) pathways). PorBC is composed of PorB and PorC homologous polypeptidic chains of about 130 amino acids. Monomeric PorB appears to have a helical structure (13) which could form a pentamer in the membrane, but there is no experimental structure available so far for this putative complex. Interestingly, PorA, PorB, PorC, and PorH have been proved to be post-translationally modified by one or several mycolate chains on dedicated serines (14, 15). Although important for membrane association, the function of this modification remains elusive for these transmembrane proteins. The pore-forming activity of PorA/PorH *in vitro* is abolished if PorA is not mycoloylated while membrane insertion of the protein does not seem to be affected (16). On the contrary, PorH and PorBC association to the membrane is largely affected by the absence of mycoloylation (17, 18). Porin mycoloylation is abolished in a mutant strain deleted for the gene encoding MytC, one of the six mycoloyltransferases described in *C. glutamicum* proving that this protein is either directly or indirectly involved in this modification. In this study, we set up an *in vitro* assay to definitely show that MytC was sufficient on its own to carry out PorA mycoloylation. By using synthetic TMM and appropriately designed analogs, we also characterized the crucial structural elements of the mycolate donor, necessary to drive the transesterification reaction catalyzed by MytC. Finally, we successfully resolved the structure of MytC trapped in its covalent intermediate state.

## Results

### MytC is able to mycoloylate PorA in vitro

**Figure 1:**
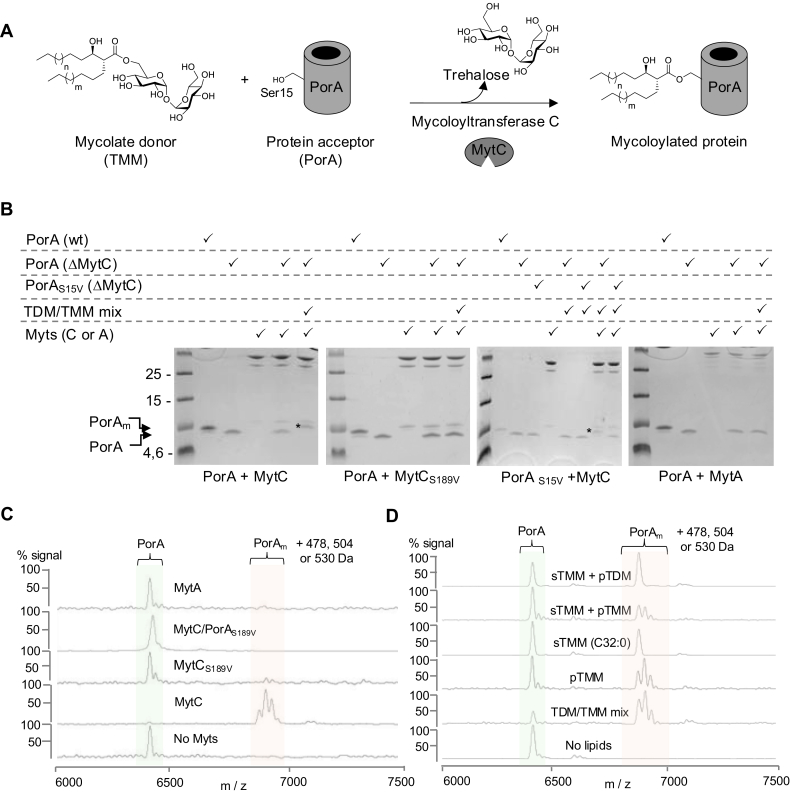
**Mycoloylation of PorA *in vitro* by purified MytC.***A*, hypothetical reaction proposed for MytC. *B*, analysis of PorA mycoloylation *in vitro* by Tricine-SDS/PAGE and visualized by Coomassie blue staining. A star is indicated next to the band corresponding to mycoloylated PorA. *C* and *D*, analysis of PorA mycoloylation *in vitro* by MALDI-TOF MS: spectra of the PorA substrate (*m/z* 6410), leading to the formation of the expected major mycoloylated products PorAm (*m/z* 6888 (C32:0), 6914 (C34:1), 6940 (C36:2)) in the presence of MytC (except if PorA_S15V_ (*m/z* = 6422) is used as a substrate) but not with MytCS189V or MytA (*C*), leading to the formation of the expected mycoloylated products PorAm (*m/z* 6888 (C32:0), 6914 (C34:1), 6940 (C36:2)) in the presence of TMM, purified from bacteria (pTMM) or synthetic (sTMM C32:0) but not TDM (*D*).

*In vivo*, deletion of mytC in *C. glutamicum* is associated with the absence of PorA, PorH, and ProtX *O*-mycoloylation indicating that MytC is clearly involved in this uncommon post-translational modification (15). In order to confirm that MytC alone is directly able to mycoloylate a protein, we decided to set up an *in vitro* test to evaluate the ability of purified MytC (Fig. S1*A*) to transfer a mycolate moiety from a lipid donor to a protein acceptor. In a first approach, we decided to test a mycomembrane lipid extract (highly enriched in TMM and TDM and hereinafter referred to as TDM/TMM mix (Fig. S1*B*), as a potential mycolate donor and purified non-mycoloylated PorA as the final acceptor of the reaction on Ser15 (14) (Fig. 1*A*). The mycoloylation of PorA was followed by SDS PAGE since mycoloylated and non-mycoloylated PorA proteins are clearly resolved on a 16% Tris-Tricine SDS-PAGE (16). As shown in Figure 1*B*, when non-mycoloylated PorA (purified from a Δ*mytC* strain) is incubated in the presence of TDM/TMM mix and MytC, an additional band (marked by a ∗ in the figure) migrating similarly to PorA purified from a wild-type strain (mycoloylated PorA) is detected. This band is absent if MytC is replaced in the reaction mixture by a variant that carries a mutated catalytic serine (S189V) excluding that the observed activity is due to a trace of contaminating protein. This band is also absent if a PorA mutant (PorA_S15V_) is used as a substrate confirming that it is the Serine 15 of PorA which is specifically modified by MytC. As expected, MytA, which is known to be involved in TMM and arabinogalactan mycoloylation (15, 19), is not able to mycoloylate PorA in similar conditions.

In order to definitively confirm that mycoloylated PorA is generated during the reaction, all samples were analyzed in parallel by mass spectrometry (Fig. 1*C*). In the presence of MytC, but not with the catalytic mutant of MytC nor with MytA, the peak corresponding to native non-mycoloylated PorA (6410 Da) shifted to a series of three main peaks with a mass difference of 478, 504 and 530 Da compared to the mass of unmodified PorA. These peaks perfectly match the natural occurrence of the three major mycolic acid species that have been described in *C. glutamicum* (C32:0, C34:1 and C36:2) (2, 14). Altogether these results unambiguously demonstrate that purified recombinant MytC is able to mycoloylate PorA when a mixture of mycomembrane lipids, *i.e.* TDM/TMM mix, is provided as mycolate donors.

### TMM, but not TDM, is a suitable mycolate donor for PorA

Only sparse information is available concerning the mechanism involved in protein mycoloylation *in vivo*, and more particularly little is known about the nature of the donor substrate. In a previous study, we showed that TMM accumulated in a Δ*mytC* strain, suggesting that this molecule could be the natural mycolate donor (15). However, TDM, the other major trehalose mycolate ester found in the cell envelope may also potentially constitute a suitable donor for MytC. Alternatively, minor glycolipids present in the mycomembrane could also be used by MytC. To address this question, we first decided to test either purified or synthetic TMM molecules as substrates in the reaction. Purified TMM (pTMM) was prepared from the TDM/TMM mix as described in the Materials and Methods section and synthetic TMM-C32:0 (sTMM) was obtained as previously described (20). Both preparations (Fig. S1*B*) of TMM are easily resuspended in an aqueous solution, and as shown in Figure 1*D*, are readily used by MytC to mycoloylate PorA. Indeed, when either a mycomembrane lipid extract (TDM/TMM mix) or purified TMM are used in the reaction, the three characteristic main peaks corresponding to mycoloylated PorA are detected in both conditions. Instead, when synthetic TMM-C32:0 is used, only a single peak corresponding to PorA modified by a C32:0 mycolate chain (Δm = 478 Da) is detected. These results clearly show that TMM is a *bonafide* donor of mycolate for protein mycoloylation *in vitro*. Because natural or synthetic TDM are mostly insoluble and very difficult to resuspend correctly in aqueous solution (21), these substrates cannot be used for an *in vitro* MytC assay. We decided to resuspend the TDM in aqueous solution by introducing synthetic TMM-C32:0 in our TDM preparations (ratio 1:1). In these conditions, we noticed that the TMM-C32:0/TDM lipid film is nicely rehydrated and does not form any visible large insoluble material. Interestingly, when this mixture is further used as a mycolate donor for PorA mycoloylation by MytC in our *in vitro* test, we clearly only observe the appearance of a single modified PorA species corresponding to the transfer of the C32:0 chain (Δm = 478 Da) but not of the expected C34:1 or C36:2 chains that would have appeared if mycolic chains from TDM were transferred (Fig. 1*D*). As a control, using a mixture of synthetic C32:0 TMM and purified TMM, we were clearly able to detect the characteristic series of three peaks corresponding to PorA species modified by all major mycolate chains (C32:0, C34:1 and C36:2) of “natural” TMM. Altogether, these results strongly suggest that TDM is probably not an efficient donor of mycolate for PorA mycoloylation in contrast to sTMM and natural TMM.

### Chemical synthesis of TMM analogs

**Figure 2:**
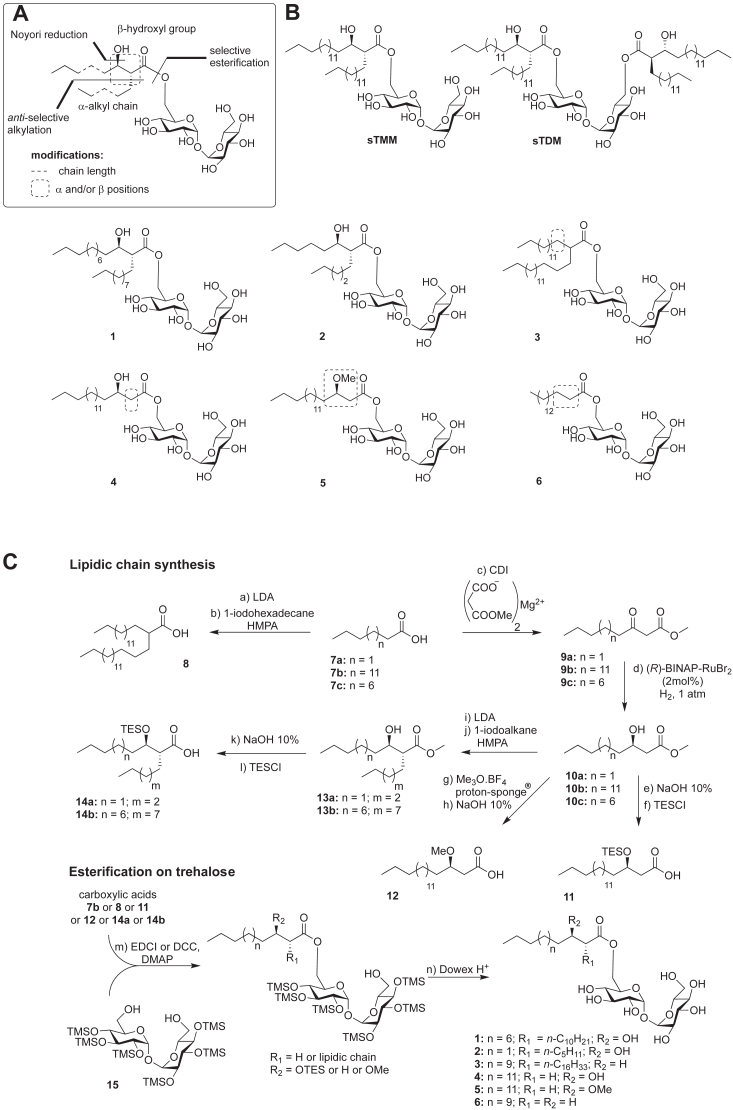
**Design and synthesis of a TMM analogs library.***A*, synthetic strategy to obtain the TMM analogs with a mycolate pattern featuring the stereochemistry of the natural compounds. *B*, synthetic “natural” TMM and TDM (C32:0) and new TMM analogs 1 to 6. *C*, brief overview of the TMM analogs 1 to 6 synthesis.

To further define the specificity of MytC toward its mycolate donors, we decided to chemically synthesize several TMM analogs with modifications introduced on the mycolate moiety (Fig. 2*A*). They were synthesized either with shorter branched acyl chains (compounds 1 (TMM-C23:0) and 2 (TMM-C13:0)), or lacking the hydroxyl group (compound 3), lacking the α-alkyl chain (compounds 4 and 5), lacking the H bond donor capacity of the hydroxyl group (compound 5), or only composed of a simple unsubstituted acyl chain (compound 6, also named trehalose monopalmitate (TMP)) (Fig. 2*B*). To date, most TMM-based reported tools contain simplified fatty acyl chain thus making their synthesis fast and efficient (22). Recently, we reported the first synthesis of a bioorthogonal TMM-based probes with the natural pattern of mycolic acid which proved to be highly efficient in labeling *C. glutamicum* (23, 24). Based on these results, we anticipated that TMM derivatives should help to decipher the enzymatic mechanism and specificity of MytC. Such compounds represent an important synthetic challenge to include the native pattern of mycolic acids featuring an OH group in β-position with an (*R*) stereochemistry and a branched α-lipid chain with an (*R*) stereochemistry and an *anti*-relationship at C_2_-C_3_ between the two β and α-substituents. Our TMM library was synthesized through two main steps: (i) preparation of the fatty acids parts based on Noyori enantioselective reduction of β-ketoester (25) and diastereoselective alkylation of the resulting β-hydroxyester using Fráter- Seebach alkylation (26, 27) and (ii) esterification on a selectively protected trehalose derivative (Fig. 2*A*). Figure 2*C* shows a brief overview of the TMM derivatives synthesis. For non-ramified and non-hydroxylated TMM analog 6, commercially available fatty palmitic acid 7b was used and esterified to trehalose 15. For TMM analog 3, carboxylic acid 8 was obtained by α-alkylation of 7b with one-iodohexadecane. Derivatives 9a-c were obtained after a two-atom homologation of their corresponding carboxylic acids 7a-c. Derivatives 9a-c were subjected to enantioselective Noyori reduction to afford the β-hydroxyesters 10a-c. Derivative 10b was then converted to carboxylic acid 11 which was then esterified with selectively protected trehalose 15 to give TMM analog 4. 10b was also treated with proton sponge and trimethyloxonium tetrafluoroborate followed by aqueous sodium hydroxide to furnish carboxylic acid 12 which was coupled to trehalose 15 to give analog 5. Compounds 10a and 10c were finally treated with LDA (lithium diisopropylamide) and iodoalkane (*i.e.* 1-iodopentane or 1-iododecane) to furnish derivatives 13a and 13b which were then converted to carboxylic acids 14a and 14b, and further esterified with trehalose 15 to give TMM analogs 1 and 2.

### The **β**-hydroxylation of the mycolic acid chain of TMM is an essential determinant of MytC specificity

To evaluate the substrate specificity of MytC, we first assessed the ability of these TMM analogs to serve as mycolate donors. For all of them, we assayed esterification (mycoloylation or acylation) of PorA in the presence of MytC in conditions strictly identical to those used in the assay described earlier. Indeed, we included for all experiments a control of PorA mycoloylation with the TDM/TMM mix in parallel. Because some analogs are not very soluble (see above for TDM), we systematically tested the different compounds alone or in combination with the TDM/TMM mix (ratio 1:1 between analog and TMM of the mix) to provide more homogeneous and comparable preparations of mycolate donor. The samples were only analyzed by mass spectrometry because the modifications of PorA by some of the very short synthetic chains would not have been resolved on SDS-PAGE. Table 1 indicates the expected increase in mass after fatty acid chain transfer catalyzed by MytC.

**Table 1:** Mass of TMM and analogs and expected Δm after fatty acid transfer onto a mycolate acceptor

TMM or analogs	TMM or analogs mass (g/mol)	TMM or analogs mass (M+Na)	Expected increase in mass after acyl or mycoloyl chain transfer on acceptors
C32:0	820	843	+478
C34:1	846	869	+504
C36:2	872	895	+530
Analog 1	694	717	+352
Analog 2 (TMM-C13:0)	554	577	+212
Analog 3	804	827	+462
Analog 4	624	647	+282
Analog 5	638	661	+296
Analog 6 (TMP)	580	603	+238

**Figure 3:**
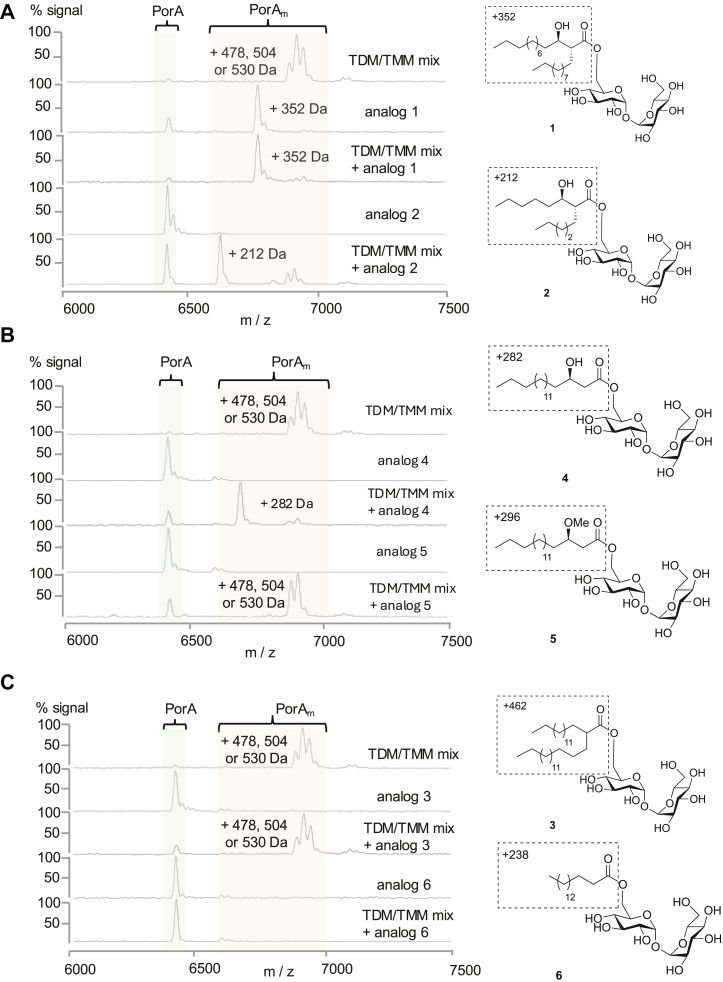
**Mycoloylation of PorA by MytC using TMM analogs 2 to 6.** Analysis of PorA mycoloylation in the presence of MytC and TMM analogs by MALDI-TOF MS. The structure of the different analogs is represented next to the corresponding spectrum and the mass of the group that is putatively transferred on PorA is indicated (region of the molecule in the box). *A*, spectra of control (without mycolate donor), analog 1, and analog 2. *B*, spectra of control (without mycolate donor), analog 4 and analog 5. *C,* Spectra of control (without mycolate donor), analog 3 and analog 6.

When PorA is incubated in the presence of analog 1 (synthetic mycolate C23:0) alone or in combination with the TDM/TMM mix, a major peak at 6758 Da is obtained, corresponding to PorA modified by a C23:0 mycoloyl chain (Δm = 352 Da) (Fig. 3*A*, analog 1, Table 1). The much shorter trehalose mycolate analog 2 (synthetic mycolate C13:0) was effectively used by MytC to transfer a C13:0 mycolate chain on PorA (peak at 6610 Da, Δm = 212 Da) only if combined with the TDM/TMM mix (Fig. 3*A*, analog 2, Table 1). This indicates that analog 2 may form micelles or other specific structures that are less accessible to MytC than those formed when combined with TDM and TMM liposomes.

Mycolate chains differ from a classical fatty acyl chain by the presence of an alkyl side chain and a hydroxyl group at the α and β positions respectively. To get insight into MytC specificity between acyl and mycoloyl chains, we assayed several analogs of TMM lacking one or both mycolic acid determinants using the same experimental approach as above. Interestingly, the absence of the α-alkyl side chain is not critical for mycoloylation by MytC since analog 4, whose main chain is similar to TMM but lacks the α-branch, is perfectly used as a substrate to acylate PorA (peak at 6691 Da, Δm = +282 Da) when provided in combination with TDM/TMM mix (Fig. 3*B*, analog 4, Table 1). On the contrary, β-hydroxylation of the mycoloyl chain is clearly essential since compound 3, an analog of TMM lacking the β-hydroxyl group, is not used as a substrate by MytC in all conditions tested (Fig. 3*C*, analog 3). Interestingly, the β-hydroxylation of the mycoloyl chain is not only required but also found sufficient to allow the transfer of a single acyl chain on PorA since a β-hydroxylated acyl trehalose is well recognized by MytC (Fig. 3*B*, analog 4) while a simple acyl trehalose is not (Fig. 3*C*, analog 6). The β-hydroxyl group of the substrate is probably crucial for hydrogen bonding (either intramolecular or with a critical residue of MytC) since compound 5, a methoxy version of analog 4 is completely unable to transfer its acyl chain either alone or in combination with the TDM/TMM mix (Fig. 3*B*, analog 5).

### TMP (analog 6) is an efficient inhibitor of MytC

As shown earlier, analogs 3, 5, and 6 (TMP) are not used by MytC as substrates since no corresponding mass modification of PorA is detected in their presence. We further tested whether these compounds are inhibitors of MytC. When 3 and 5 are used in combination with the TDM/TMM mix, we still observe a modification of PorA by the different mycolate chains of the TMM indicating that they do not interefere with the reaction. This is not the case for TMP that seems to inhibit the reaction (Fig. 3*C*, analog 6). To confirm this hypothesis, we incubated PorA and MytC with increasing amounts of TMP with a fixed concentration of TDM/TMM mix (2 mM). As expected, the characteristic peaks corresponding to mycoloylated PorA progressively disappeared and the reaction is completely inhibited at 0.5 and 1 mM of TMP (Fig. S2). We do not know at which step PorA mycoloylation is inhibited by TMP (formation of the mycoloyl-enzyme intermediate or transesterification on PorA, see Fig. 1*A*), but it is clear that TMP, although recognized by MytC, is not in a correct interaction with the catalytic site of MytC to allow the transfer of its acyl chain on PorA.

### Both TMM-C13:0 (analog 2) and TMP (analog 6) are efficient substrates for MytA-catalyzed trehalose mycoloylation

**Figure 4:**
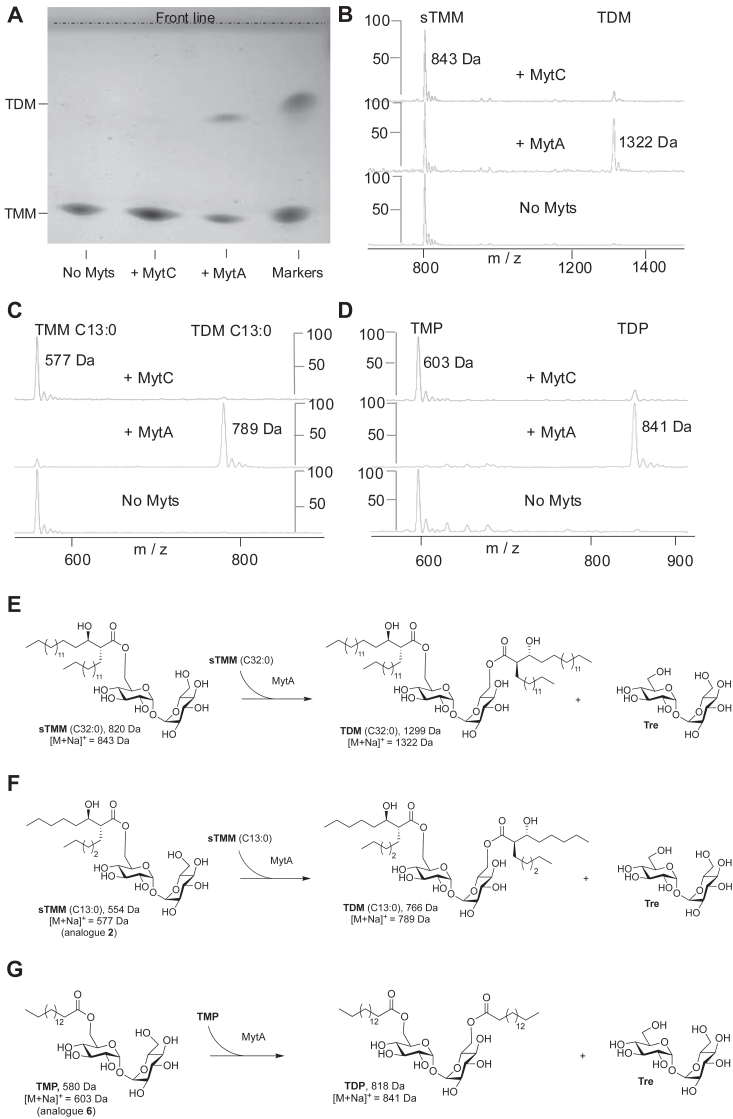
**Mycoloylation of TMM and analogs by MytC and MytA.***A*, analysis of sTMM C32:0 mycoloylation in the presence of MytC and MytA by TLC. *B*, analysis of sTMM mycoloylation in the presence of MytC and MytA by MALDI-TOF MS (sTMM C32:0 substrate: *m/z* 843 and TDM expected product: *m/z* 1322). *C*, analysis of TMM analog 2 mycoloylation in the presence of MytC and MytA by MALDI-TOF MS of the analog 2 substrate (*m/z* 577). Expected mass of the TDM analog 2 product: *m/z* 789. *D*, analysis of TMM analog 6 mycoloylation in the presence of MytC and MytA by MALDI-TOF MS of the analog 6 substrate (*m/z* 603). Expected mass of the TDM analog 6 product (TDP): *m/z* 841. *E*–*G*, expected mycoloylation reactions of TMM and analogs 2 and 6. The scale represents the % of the signal.

*C. glutamicum* mycoloyltransferases have been shown to transfer mycolate chains on various cell wall acceptors such as trehalose and arabinose in order to build the mycomembrane (6). *In vivo*, the deletion of MytA has dramatic effects on the synthesis of TDM while the deletion of MytC is much less deleterious in glycolipid synthesis (8). *In vitro*, mycoloyltransferases of Mycobacteria (Ag85) have been shown to use TMM as a donor substrate to mycoloylate another molecule of TMM giving rise to TDM (28). This reaction has been further studied by using various alternative substrates to set up fluoro- or colorimetric assays (29, 30) suitable for high throughput screening. Here we tested the activity of MytA and MytC for their ability to synthesize TDM from synthetic TMM *in vitro* (Fig. 4*E*). Myts proteins were incubated in the presence of synthetic TMM and the putative formation of TDM was detected by thin layer chromatography (TLC) after sulfuric acid staining or by mass spectrometry. After 3 h of incubation, a small amount of TDM is detected on TLC when the reaction was performed in the presence of MytA but not in the presence of MytC (Fig. 4*A*). This result is also confirmed by mass spectrometry. In the presence of MytA, a peak corresponding to TDM (1322 Da) is clearly detected while only a very small one is detected when MytC is added into the reaction (Fig. 4*B*). These results suggest that, unlike MytA, MytC does not use efficiently TMM as an acceptor molecule in this reaction. Because we showed that MytC displayed a very strict specificity regarding the mycoloyl and the acyl groups that are transferred on PorA, we checked whether MytA has the same requirements in its ability to mycoloylate trehalose. For this, we incubated MytA (or MytC as a control) in the presence of analog 2 (TMM C13:0) or 6 (TMP). As shown in Figure 4, *C* and *D*, both compounds are efficient substrates of MytA which is able to transfer either a mycolate or an acyl chain from TMM C13:0 or TMP respectively to another molecule of these analogs to form TDM C13:0 and Trehalose dipalmitate (TDP) (see reactions in Fig. 4, *F* and *G*)). For MytC, only very small peaks corresponding to the product of the reaction are detected, similarly to what is observed when authentic TMM-C32:0 is used as a substrate. This result indicates that MytA, unlike MytC, is not able to discriminate between an acyl and a mycoloyl chain *in vitro*.

### TMM-C13:0 (analog 2) traps MytC in its mycoloyl-enzyme transition covalent intermediate

**Figure 5:**
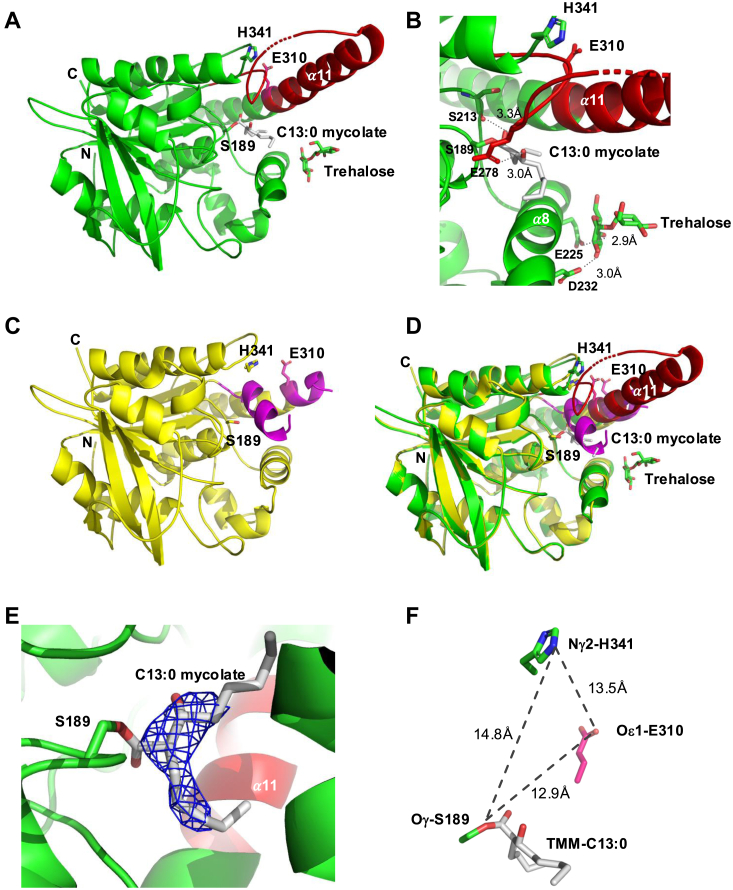
**Crystal structure of the MytC-acyl enzyme.***A*, ribbon presentation of of the MytC-acyl enzyme (MytC in complex with TMM-C13:0, this work, pdbcode:8QHF). The *N* and *C* termini are labelled as well as the residues composing the catalytic triad (S189, E310 and H341). Covalently bound C13:0 mycolate and trehalose are in sticks. The region in *red* was mostly disordered in the crystal structure of apo-MytC. *B*, Zoom of the catalytic site bound to the C13:0 mycolate chain. MytC polar interactions with C13:0 mycolate chain and trehalose are indicated as dashed lines. *C*, structure of apo-MytC (pdbcode: 4H18). The helix blocking the active site and part of the α11 helix are in *pink*. *D*, superposition of the MytC-acyl enzyme and apo-MytC crystal structures. *E*, polder map contoured at a 3s level (the TMM-C13:0 was not included in the calculation of the map). *F*, relative positions of the catalytic residues in the MytC-acyl enzyme.

The resolution of enzyme structures in the presence of mycolic acid-like substrate has significantly increased the understanding of the reaction mechanism of mycobacterial mycoloyltransferases. For instance, Ag85C has been crystallized in the presence of diethyl p-nitrophenyl phosphate (31), octyl-glucoside (32), cyclophostin analog CyC_8β_ (33), and more recently with tetrahydroxylipstatin (THL) yielding a covalent enzyme-inhibitor complex (34). Based on these structures a convincing catalytic mechanism consisting of two successive mycolate transfer reactions could be proposed. Here, to further explore the mechanism and the specificity of protein mycoloylation, we took profit of our TMM analog library to obtain crystals from MytC trapped in its acyl-enzyme state. We tested both the TMM-C13:0 (analog 2), a water-soluble analog of TMM and TMP (analog 6), the previously characterized inhibitor of the reaction. We obtained diffracting crystals of MytC with both compounds and their structure was solved by molecular replacement. TMP was not detected in the crystal and the structure of MytC determined in these conditions was identical to the one obtained for the apoenzyme. The structure of the crystal of MytC incubated with TMM-C13:0 was solved at 2.69 Å (PDB accession code 8QHF) (Fig. 5, *A* and *B*). The protein crystallized in the very rare space group F23 and the asymmetric unit contained one copy of MytC with a solvent content of 49% (Table 2). This space group was not obtained with the apo-form of MytC nor with MytC homologues. They are only three structures related to MytC that crystallized in F23 space group from the 152 proteins deposited in the PDB data bank. Two are the gamma lactamases (1HKH and 1HL7) and the other one is a 3-dehydroquinate dehydratase (2C4W). The Polder map shows that S189 Oγ atom forms an ester bond with the carbonyl of the analog (Fig. 5*E*). This structure will be further referred to as the MytC-acyl enzyme (MytC with catalytic S189 esterified). Electron density in the MytC-acyl enzyme was well defined for residues 30 to 363, except for the segment between 283 to 287. The meromycolic chain of the mycolate C13:0 is directed towards the exit of the active site cave, while the α-chain is more deeply buried and establishes hydrophobic interactions with residues of MytC α11 helix (Fig. S3*A*). The β-hydroxyl group, a determinant for PorA modification by MytC, does not establish polar interactions with MytC. Modeling of full-length alkyl chains of a TMM substrate (C32:0) onto the MytC-acyl structure shows that these could be comfortably accommodated by the MytC active site canyon (Fig. S3, *A* and *B*). Interestingly, comparison of the active sites of MytC-acyl enzyme and Ag85C-THL (5VNS) (Fig. S3*C*) shows that, although the main chain atoms of the catalytic serines (S189 and S124 respectively) superpose exactly, the dihedral χ1 angles of the side chains differ by about 60°, and the ester-carbonyl of the two covalent intermediates are in totally opposite directions. While the carbonyl in the tetrahydrolipstatin intermediate points towards a putative oxyanion hole, the equivalent carbonyl of MytC-acyl enzyme does not establish polar interactions with the enzyme. The β-OH groups occupy about the same position in both enzymes despite the opposite configuration at the carbon C-3 (Fig. S3*D*).

**Table 2:** Data collection and refinement statistics of the MytC-acyl enzyme

Data collection and refinement	Crystal MytC-TMM C13:0
**X-ray source**	PROXIMA2 (2021/10/13)
**Wavelength (Å)**	0.98013
**Unit-cell (Å, °)**	168.12, 168.12, 168.12, 90.0, 90.0, 90.0
**Space group**	F23
**Resolution limits (Å)**	48.53 - 2.69 (2.96 – 2.69)
**Total reflections**	107266 (25541)
**Unique reflections**	21304 (5234)
**Completeness (%)**	99.39 (97.85)
**Redundancy**	5.0 (4.9)
**Mean I/σ (I)**	7.7 (0.9)
**R-merge (%)**	17.9 (187.6)
**R-pim**	8.9 (94.8)
**R-meas (%)**	20.1 (210.7)
**CC (1/2)**	99.4 (27.8)
**Wilson B factor (Å**^**2**^**)**	68.17
**Reflections used in refinement**	10492
**Reflections used for R-free**	553
**R/R**_**free**_**(%)**	25.3/27.9
**Number of nonhydrogen atoms (protein/TMM-C13:0/Trehalose/SO4/Cl/H2O)**	2512/15/23/5/4/31
**R.M.S.D. Bonds (Å)/angles (°)**	0.007/0.89
**Average B factor (Å**^**2**^**) (total/protein/TMM-C13:0/Trehalose/SO4/Cl/H2O)**	85.0/83/78.2/101.6/145/98.3/71.2
**Ramachandran (%)** **(Favored/Allowed/Outliers)**	96.3/3.7/0
**Rotamer outliers (%)**	2.3
**Clashscore**	3.63
**PDB**	8QHF

Statistics for the highest-resolution shell are shown in brackets.

The MytC catalytic triad residues S189, H341, and E310 are clearly in an inactive configuration: the H341 Nδ is at a distance of 14.8 Å from the S189 Oγ and 13.5 Å from the E310 Oε1 (Fig. 5*F*). As shown in Figure 5, *C* and *D*, the segment between residues 271 to 311 undergoes a major conformational change in the MytC-acyl enzyme compared to the apo enzyme (PDB accession code 4H18, (15)). In the apoform, a small helix (η3) contained between residues 270 to 280, is positioned between S189, H341 and E310, blocking the active site pocket. This helix unfolds in the MytC-acyl enzyme structure to form an irregular loop, becoming part of the active site wall, thereby providing space for the binding of the α-alkyl moiety of the mycolate chain C13:0. Residues 291 to 304, disordered in the apo form, have a well-defined structure in MytC-acyl enzyme, forming an extension of the α11 helix that contacts the C13:0 alkyl chain.

A trehalose moiety was identified on the surface of MytC, near the entrance of the active site groove. One of the trehalose glucose moieties makes polar contacts with the side chains of residues E225 and D232 of helix α8 (Fig. S4). These residues are not conserved among mycoloyltransferases and the biological significance of this binding site remains unclear.

To further investigate the flexibility of the active site region, we compared our MytC-acyl enzyme structure with the Alphafold (AF)-model of MytC present in the AF database (Fig. S5*A*). The pLDDT score of the AF model is very high (>90) for most of the sequence and is around 70 for the region between residues 272 and 308. The AF-model superposes very well onto the MytC-acyl crystal structure (rmsd of 0.29 Å for 266 Cα positions). The AF model also has a fully formed α11 helix which more pronouncedly bends towards the active site pocket as compared to the MytC-acyl enzyme structure. Interestingly, the AF-model proposes a conformation for the loop between residues 336 and 343 that is different from that of the MytC-acyl enzyme structure. This loop swings into the active site pocket and positions His341 between Ser189 and Glu310. Due to the conformational change of this loop and the bending of the α11 helix, the catalytic triad of the AF model is in a catalytically competent configuration (Fig. S5*B*).

### Modeling of MytC-PorA interaction by Alphafold

**Figure 6:**
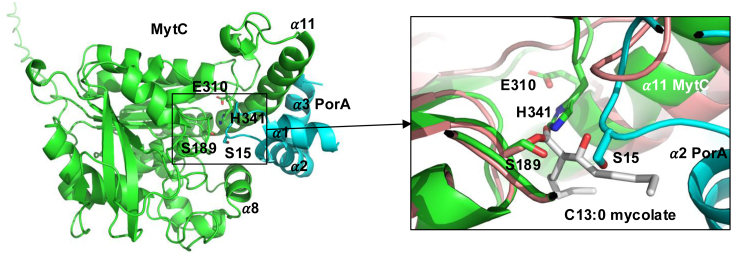
**AlphaFold model of the MytC-PorA complex**. MytC is shown in *green* and PorA in *light blue*. The catalytic triad residues of MytC (S189, E310, and H341) and the S15 of PorA that becomes mycoylated are labelled. Inset: zoom of the active site showing the superposition of the MytC-acyl crystal structure (salmon) and the AF model of MytC-PorA (same colors as in the *left* part).

For the moment, there is no experimental structural information available concerning the PorA-MytC interaction. We therefore constructed a model of the MytC-PorA complex using AlphaFold-assembly. The model of the PorA protein alone is of good quality, proposing two helical segments. Interestingly, the targeted Ser15 is situated in a very short linker that connects the two helices (Fig. S6). In the MytC-PorA complex, PorA forms a helical hook-shaped structure that clips between helices α8 and α11 into the active site of MytC (Figs. 6, and S7). Interestingly, the mycoloylation target residue of PorA (S15) is located in a loop that connects two helices of PorA. This loop sits deeply into the active site of MytC facing the catalytic serine (Fig. 6). When superposing onto the MytC-acyl structure, the Ser15 Oγ is at 7 Å from the carbonyl carbon atom of the mycolate C13:0 (inset Fig. 6). As for the Alphafold (AF)-model of apo-MytC, the catalytic residues Ser189, His341, and Glu310 were in an active configuration in the AF-model of the MytC-PorA complex (Figs. S5 and S7). The superposition (inset Fig. 6) also suggests that the β-hydroxyl group of the C13:0 mycolate chain could engage a polar interaction with PorA which might explain why its presence is required for the mycoylate transfer on the protein.

## Discussion

Mycoloylation of porins in *C. glutamicum* has been discovered in 2010 (14) and later proved to be dependent, *in vivo*, on *mytC* expression (15). Here we set up an *in vitro* test showing that purified MytC is indeed able to mycoloylate PorA provided TMM is added to the reaction mixture. Importantly, another main mycoloyltransferase in *C. glutamicum*, MytA, is not able to mycoloylate PorA in identical experimental conditions. This result definitely proves that MytC is in fact the mycoloyltransferase directly involved in this atypical post-translational modification. It also indicates that MytC is able to recognize its acceptor substrate without the assistance of any other proteins although we cannot exclude that the involvement of other partners may improve the efficiency of the process or may be required for the mycoloylation of other proteins such as PorB and PorC which were not tested in this study. By using different synthetic analogs of TMM, we explored the specificity of MytC towards its mycolate donor and more particularly probed the importance of the mycolic acid motif. Our results clearly showed that the presence of the β−hydroxyl group of the meromycolate chain, a hallmark of all mycolic acids, is a key determinant of MytC specificity while the presence of the α-chain is not mandatory. This finding is particularly interesting if compared to MytA which, in contrast, does not have this strict specificity. Indeed, MytA is perfectly able to catalyze *in vitro* the transfer of a palmitoyl group from analog 6 (TMP) onto another TMP to form di-*O*-palmytoylated trehalose (an acyl analog of TDM) (Fig. 4, *D* and *G*). MytA and MytC are therefore distinct mycoloyltransferases that evolved in their specificity both for donor and acceptor substrates. Interestingly, all these *in vitro* observations are consistent and may sustain *in vivo* data of the literature showing that deletion of *mytA*, but not of *mytC*, has a very dramatic effect on TDM synthesis (7). Conversely, deletion of *mytC* has been described to completely abolish PorA mycoloylation which can only be restored by ectopic expression of *mytC* but not of *mytA* (15). Concerning the promiscuity of MytA towards its mycolate donor, it is important to notice that acyl-TMM molecules have been detected in *C. glutamicum* according to two independent lipidomic studies (35, 36). In the light of our results, it is therefore tempting to speculate that MytA could be responsible *in vivo* for their synthesis from acyl-trehalose molecules. In contrast, the strict specificity of MytC towards its mycolate donor seems essential to clearly distinguish protein mycoloylation from the *bona fide* Lgt/Lnt protein acylation machinery. The coexistence of two distinct lipidation pathways in *C. glutamicum* may suggest that each of them is dedicated to two independent subsets of proteins that may require distinct modification to adopt their active conformation or to be sorted to their final destination. In this context, it is tempting to speculate that MytC has evolved in order to avoid any unfortunate acylation of proteins that otherwise would lead to their mis-localization and/or mis-folding and may be deleterious for the cell. Finally, our finding that MytC, but not MytA, is very specific for β−hydroxylated acyl chains somehow support the choice of acyl-trehalose probes that have been developed recently in order to label the mycomembrane (37) but not those intended to identify the mycoloylome by metabolic labeling (24, 38).

The MytC apoform structure (15) revealed that its catalytic triad adopted an inactive conformation and that its active site was blocked by a small helix. The ensemble of MytC structures and models highlights the highly flexible nature of its active site region, capable of adopting different conformations of the catalytic machinery. Upon formation of the acyl-intermediate, the blocking helix unfolded and was displaced from the active site pocket, providing space for the covalently bound acyl moiety. The H-bonding network of the catalytic triad however remained disrupted in the acyl-form. We hypothesize that this inactive form of the MytC-acyl enzyme is a temporary conformational that occurs before the arrival of its acceptor substrate PorA. This probably represents a strategy to prevent futile hydrolysis of the reaction intermediate, as has been suggested for other mycoloyltransferases (39). The considerable flexibility of the MytC active site region is further illustrated by the restructuring of the residues 280 to 305, which transit from a disordered state in apo-MytC to an elongation of the α11 helix in the MytC-acyl enzyme. The towering position of this helix next to the active site pocket suggests that the α11 helix could be involved in interaction with the PorA substrate. Alphafold modeling of the MytC-PorA complex endorses this hypothesis. In this model, the PorA protein forms a very amphiphilic helical bundle whose hydrophobic surface wraps around a hydrophobic patch of the helix α11 of MytC. The putative MytC binding site for PorA (residues 300–311) is disordered in the apo-form of the enzyme but, elongates the α11-helix in the structure of the acyl-intermediate. The putative PorA binding site overlaps with the regions of MytA and Ag85C involved in binding of the acceptor TMM to synthesize TDM, suggesting that this region has evolved in MytC to accommodate PorA instead of TMM. Superposition of the MytC-PorA complex onto MytA, shows that two of the PorA helices overlap with two helices at the *N*-terminus of MytA that also blocked the active site region of the latter enzyme. Superposition of MytC-PorA onto the acyl-MytC also shows that the β-hydroxyl group could interact with PorA (inset Fig. 6), explaining why this group might be a discriminating determinant for PorA mycoylation. The model also convincingly positions the serine 15 mycoloylation target facing the carbonyl of mycolate C13:0 bound to MytC.

Previous studies on mycoloyltransferases have provided important structural data but none of these included compounds that had the exact pattern of natural mycolic acids. For instance, a structure of the mycoloyltransferase Ag85C-acyl-enzyme was obtained in an elegant way using THL, a versatile lipid esterase covalent inhibitor. Indeed, this natural compound features two chiral carbons that mimic the core attributes of the mycolic acid. The 2-alkyl, 3-hydroxy fatty acid moiety has however an inverted configuration concerning the asymmetric carbons of the natural mycolic acids. While the two THL carbons of interest have (2*S*, 3*S*) configurations, the natural mycolic acids have an opposite (*R*) configuration at the two chiral carbons, C2 and C3 (40). Furthermore, THL carries a voluminous peptidyl side arm which is absent in mycolic acids. The structure of the Ag85C-acyl-enzyme, combined with molecular dynamics, provided an interesting hypothesis regarding the mechanism of the transesterification reaction: (i) the binding of the incoming acceptor molecule could drive Ag85C to form an active conformation and (ii) the β-hydroxy of mycolic acid would directly or indirectly activate the incoming nucleophile. When comparing MytC acylated with a C13:0 mycolate carrying the native carbon configurations with the Ag85C-acyl-enzyme, we noticed that their ester moieties bound to the catalytic serine are in opposite orientations (Fig. S3*D*). This implies a significantly different positioning of the electrophilic carbonyl to be attacked by the incoming nucleophilic alcohol of the acceptor. Interestingly, despite the opposite configuration of the C3, the β-hydroxyl group is accommodated in a relatively similar way. Consequently, the two alkyl chains do not project in the same direction in the two structures. Here, the α-chain is perfectly positioned in the hydrophobic pocket, while the meromycolic chain remains in another more interfacial cleft, directed towards the exit of the active site cave. This is a clear difference from the previously proposed model based on the Ag85C-THL structure (41) and that does not necessarily exclude an interfacial mechanism “scooting mechanism” as proposed previously.

**Figure 7:**
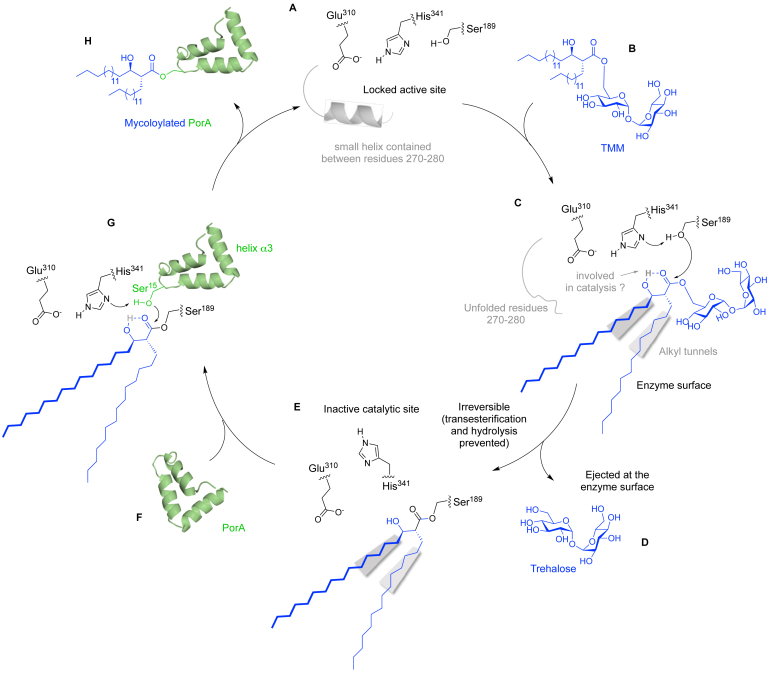
**Proposed structure-based catalytic cycle of MytC.***A*, active site of the apo-enzyme locked by a small helix. *B*, entrance of the TMM mycolate donor. *C*, nucleophilic attack of the catalytic serine 189 that forms the first tetrahedral intermediate. *D*, trehalose is ejected at the surface of MytC. *E*, acyl-enzyme intermediate with histidine 341 in an inactive position. The blocking helix unfolds and provides space for the covalently bound acyl moiety. *F*, the entrance of the PorA mycolate acceptor. *G*, acyl-enzyme intermediate with histidine 341 in an active position. Nucleophilic attack of serine 15 of PorA that forms the second tetrahedral intermediate. *H*, mycoloylated PorA is ejected from the catalytic site.

We finally extract from our biochemical and structural studies a hypothetical mechanism of the MytC-catalyzed reaction (Fig. 7). In the resting state the active site pocket of the apo-enzyme is locked by a small helix (η3) which unfolds upon entrance of the TMM mycolate donor into the active site. After formation of the intermediate ester, the crucial β-hydroxyl group could either interact with the catalytic triad or activate the carbonyl for nucleophilic attack through intramolecular hydrogen-bond formation. The inactive configuration of the catalytic triad protects the intermediate from being hydrolyzed. Binding of the PorA mycolate acceptor restores the active configuration of the catalytic triad and through interactions with the α8 and α11 helices of MytC, inserts its serine 15 in the catalytic site for the subsequent transesterification.

## Conclusion

Lipoproteins are essential in bacteria where they represent between 2% and 3% of total proteins and are involved in various functions such as cell physiology, nutrients influx, drug efflux or pathogenicity. Whereas in *Corynebacteriales*, acylated proteins have been well characterized, the protein *O*-mycoloylation has only recently been discovered in *C. glutamicum* as a new type of protein lipidation whose exact function in cell envelope organization is puzzling. In this study, the MytC-mycolate acyl-enzyme structure and the structure-activity relationship data obtained from a collection of synthetic TMM analogs clearly show a unique mechanism and specificity within the mycoloyltransferases family. In particular, the role of the β-hydroxyl group of mycolic acid, which had previously been suggested as important in the Ag85C mechanism, is here unambiguously demonstrated as the key determinant to specifically individualize acylation and mycoloylation pathways in the cell. We also observed the presence of an efficient enzyme-locking system to avoid hydrolysis processes in this transesterification reaction. Altogether, these findings provide additional knowledges in our understanding of the specificity and mechanism of the mycoloyltransferases whose apparent redundancy is still not completely understood in *Corynebacteriales*. These results could be useful in designing specific inhibitors for each individual mycoloyltransferases which could be interesting targets for innovative therapeutic approaches.

## Experimental procedures

### Bacterial strains and growth conditions

All *C. glutamicum* strains used in this study are derivatives of the strain ATCC 13032 RES167. *C. glutamicum* ATCC13032 RES167 was grown in brain heart infusion (BHI) medium with shaking (220 rpm) at 30 °C.

### Plasmids construction

The plasmids used in this study for protein expression were constructed previously. MytA_his_ encoding gene was cloned in pCGL482 under its own promoter and signal sequence (42) and introduced in *C. glutamicum ΔmytA* strain. MytC_his_ encoding gene was cloned in pCGL482 under its own promoter and signal sequence (15) and introduced in *C. glutamicum* ATCC 13032 Δ*mytC* strain. MytC_his_ is highly expressed (5 mg L^−1^) and partly secreted in the culture supernatant or localized in the cell envelope. PorA_his_ encoding gene was cloned in pXMJ19 under a *tac* promoter (16) and introduced in *C. glutamicum* ATCC 13032 Δ*mytC* strain. PorA_his_ is moderately expressed and essentially found in the cell envelope.

### Expression and purification of MytC_his_, MytA_his_ and PorA_his_

#### Purification of MytA_his_

For large-scale purification of MytA_his_, cells (*C. glutamicum* Δ*mytA* strain transformed with *p*CGL482-*mytAhis*) from 1.2 L overnight cultures (BHI + Cam 6 μg/ml, 30 °C) were removed by centrifugation at 7000*g* at 4 °C. Proteins were then precipitated from the supernatant by adding ammonium sulfate to 70% saturation. Incubation was carried out for 1 h at 4 °C with constant shaking. After centrifugation at 7000*g* for 15 min at 4 °C, the protein-containing pellet was resuspended in 60 ml of Tris 25 mM buffer pH 7.5, NaCl 200 mM (purification buffer). The solution was dialyzed for 24 h at 4 °C against the purification buffer (Spectrum, Spectra/Por MWCO 10 kDa) before loading on a 1 ml Ni-nitrilotriacetic acid (NTA) column. The flow through was recovered and loaded one more time on the column. Proteins weakly associated with the Ni-NTA-resin were washed off by running 40 ml of purification buffer. Elution was finally performed with three column volumes of purification buffer containing imidazole at 250 mM. Fractions containing MytA_his_ were dialyzed in 10 mM phosphate buffer pH 8.0 and 10 mM NaCl. The purification was controlled by running 12% SDS PAGE (see Fig. S1*A*) gels after ammonium sulfate, Ni-NTA, and dialysis. The concentration of MytA_his_ (ε_280_ = 140,385 M^−1^ cm^−1^, 66,982 Da) was determined using absorbance at 280 nm.

#### Purification of MytC_his_

For large-scale purification, Δ*mytC* (*mytC*_*his*_) cells were recovered from 1.2 L overnight overnight culture by centrifugation at 6000 rpm at 10 °C. Cell pellet was washed with 300 ml of 50 mM Tris-HCl, pH 8.0, resuspended in 15 ml 25 mM phosphate buffer, pH 8.0 containing 1% Triton X100 and 0.1 mg/ml AEBSF (4-(2-Aminoethyl) benzenesulfonyl fluoride hydrochloride) and left at 4 °C for 2 h with continuous shaking. The detergent extract, enriched with cell wall proteins, was collected by centrifugation at 4000*g* for 10 min at 4 °C, diluted in 25 mM phosphate buffer (pH 8.0) to reach a Triton X-100 final concentration of 0.5%, supplemented with 10 mM imidazole and added to 0.5 ml of Ni-NTA resin (Macherey-Nalgen) pre-washed with 25 mM phosphate buffer, pH 8.0, Triton X-100 0.5%, 10 mM imidazole. After 2 h incubation at 4 °C, the resin was washed with the purification buffer (10 ml 25 mM phosphate buffer) containing 0.5% Triton and 10 mM imidazole. A second step of washing was performed in the same buffer except that Triton X100 is replaced by 0.05% Dodecylmaltoside (DDM). Elution of the protein was finally performed with four resin volumes of purification buffer containing 0.05% DDM and imidazole at 250 mM. Fractions 1 to 4 were then dialyzed in 500 ml of 10 mM Tris, 10 mM NaCl (pH 8.0) (reaction buffer). The whole purification procedure (Ni-NTA) was always done on the same day, and aliquots of MytC_his_ were concentrated and conserved at −80 °C. The purification was controlled by 12% SDS-PAGE gels (see Fig. S1*A*) after ammonium sulfate and Ni-NTA steps. Protein quantification was performed by using a Nanodrop spectrophotometer after the final dialysis (ε_280_ = 86,525 M^−1^ cm^−1^, 37,451 Da).

#### Purification of PorA_his_

Recombinant PorA protein was purified either from WT or Δ*mytC* strain according to Issa *et al.* (17). Proteins were dialyzed in Tris-HCl 10 mM, NaCl 10 mM, pH 8.0 buffer and aliquots were stored at −20 °C. The purity and homogeneity of proteins were analyzed on 16% Tricine SDS-PAGE (43) (Fig. S1*A*).

### PorA mycoloylation assay

The protein mycoloylation activity of MytC was evaluated by using non-mycoloylated PorA_his_ as a substrate. PorA_his_ mycoloylation was assessed by SDS PAGE and MALDI TOF analysis. A standard reaction mixture (30 μl) consisting of 6 μM PorAhis, 5 μM MytC, 1 mM of mycolate donor (TDM/TMM mix, synthetic TMM or TMM analogs) was incubated at 37 °C for 3 h in reaction buffer (Tris-HCl 10 mM, NaCl 10 mM, pH 8.0). The reaction was stopped either by adding loading buffer (30 μl) before electrophoresis analysis on SDS Tricine gels or, by adding the matrix for MALDI-TOF analysis (sinapinic acid, 20 mg/ml in H_2_O/CH_3_CN 1:1).

### Trehalose mycoloylation assay

A standard reaction mixture (50 μl) consisting of 5 μM MytC or MytA, 1 mM of sTMM was incubated at 37 °C for 3 h in reaction buffer (Tris-HCl 10 mM NaCl 10 mM, pH 8.0). The reaction was stopped at −20 °C. Aliquots were then mixed with the matrix for MALDI-TOF analysis (6-aza-2-thiothymine, 10 mg/ml in H_2_O/CH_3_CN 1/1) or loaded on TLC and SDS-PAGE. Similar reactions were run by using TMM C13:0 and TMP instead of sTMM at 1 mM final concentration.

### Preparation of TDM/TMM mix and purification of TMM

Trehalose mycolates were purified from membrane vesicles secreted by the strain ATCC 13032 Δ*aftB* (44). Bacteria were grown in BHI (600 ml) overnight and the culture supernatant was recovered after two successive centrifugations (6000*g*, 15 min at 4 °C). Vesicles were isolated by ultracentrifugation (35,000 rpm for 2 h at 4 °C in a 45 Ti rotor) and resuspended in 20 ml of Hepes 25 mM pH 7.4 at 4 °C and membrane lipids extracted by addition of 20 ml chloroform and 40 ml methanol. After 2 h incubation, 20 ml of chloroform and 20 ml of H_2_O were added and the solution further incubated for 24 h. The chloroform phase was recovered and evaporated. Lipids were resuspended in 2 ml and stored at −20 °C. At this stage the lipid extract (TDM/TMM mix) is mainly enriched in TMM and TDM as shown in Fig. S1.

For TMM purification, the TDM/TMM mix was subjected to a preparative thin layer chromatography on a silica plate using a solvent mixture composed of chloroform/methanol/H_2_O 34:15:2. TMM was recovered by scratching the plate at the dedicated migration position of the lipid by comparison with a reference plate migrating simultaneously and revealed by vaporating a mixture of ethanol and sulfuric acid (90:10) on the plate which is then heated at 130 °C until appearance of the bands (see Fig. S1*B*).

### Lipid analysis by thin-layer chromatography

Lipids were analyzed by TLC as described by Dietrich *et al.* (42). Separation was performed on silica gel-coated plates (Durasil-25, 0.25-mm thickness; Macherey-Nagel) developed with CHCl_3_-CH_3_OH-H_2_O (65:25:4 [vol/vol/vol]). Lipids were detected by bathing plates with a solution of 10% H_2_SO_4_/90% ethanol, followed by heating.

### Mass spectrometry MALDI-TOF analysis

MALDI-TOF spectra were acquired on an Axima performance mass spectrometer (Shimadzu corporation) equipped with a pulsed nitrogen laser emitting at 337 nm and an accelerating voltage of 20 kV. All spectra were acquired in the positive linear or reflectron mode.

For MytC *in vitro* assay, a mixture of 1 μl of sample and 1 μl of sinapinic acid matrix solution (20 mg/ml in H_2_O:CH_3_CN 1:1, TFA 0.1%) was deposited onto the MALDI plate and allowed to dry in air. External mass calibration was performed thanks to SpheriCal Aqua Protein Low kit (Sigma).

For the trehalose mycoloylation *in vitro* assay, a mixture of 1 μl of sample and 1 μl of 6-aza-2-thiothymine (ATT) matrix solution (10 mg/ml in H_2_O:CH_3_CN 1:1) were deposited onto the MALDI plate and allowed to air dry. External calibration was performed with pepmix XT kit (Fisher Scientific).

### TMM analogs synthesis

Synthetic details and characterization of all previously unreported compounds are provided in supplementary materials.

### MytC crystallization with TMM analogs and resolution of the protein structures

The MytC (126.5 μM) and TMM C13:0 or TMP (1.265 mM) mixtures were incubated at room temperature for 1 h and then co-crystallized by sitting-drop vapor diffusion at 18 °C (291 °K) from a 0.1 ml:0.1 ml mixture of protein complex solution with crystallization solutions with a TTP LabTech's mosquito Crystal robot. Crystals of the MytC-TMM C13:0 complex were obtained using a crystallization solution composed of 0.1 M sodium chloride, 1.4 M ammonium sulfate, 0.1 M Hepes pH 7.1. The MytC-TMM C13:0 crystals were transferred to a solution composed of 1.5 M ammonium sulfate, 0.1 M sodium chloride, 0.1 M Hepes pH 7.1 and 23% of glycerol incremented with 0.5 mM TMM-C13:0 before flash-cooled in liquid nitrogen. The crystallization solution for the MytC-TMP crystals contained 0.2 M magnesium chloride, 0.1 M Tris pH 8.5 and 32% (w/v) PEG 4000. For diffraction experiments the MytC-TMP crystals were transferred to the crystallisation solution incremented with 15% glycerol and 0.5 mM TMP before flash-cooled in liquid nitrogen. Diffraction data were recorded on beam line Proxima2 (synchrotron SOLEIL, France) and were processed using the XDS package (45). The structure was determined by the molecular replacement method using the structure of apo-MytC as model (PDB accession code 4H18) and the program Molrep (46) implemented in ccp4 (47). The model was further improved by iterative cycles of manual rebuilding using COOT (48) and refinement using BUSTER program (49). Statistics for data collection and refinement are summarized in Table 2. A polder map without the TMM C13:0 was generated around the catalytic residue Ser189, using Phenix (50). The polder map is an omit map which excludes the bulk solvent around the omitted region. Weak electron densities, which can be obscured by bulk solvent, may therefore become visible (Fig. 5*E*). A model of MytC in complex with full-length alkyl chains of a TMM substrate was made by manually extending the alkyl chains of TMM C13:0 using COOT and then performing a geometric minimization as implemented in the Phenix program. The atomic coordinates (and structure factors) or the MytC-C13:0 mycolate complex have been deposited into the Brookhaven Protein Data Bank under the accession number 8QHF.

### Alphafold models

The models of PorA (Fig. S6) and of MytC-PorA complex (Fig. S7), were obtained using Alphafold-Multimer v2.3 as implemented in I2BC (51, 52, 53).

## Data availability

The atomic coordinates of the new structure described in this article have been deposited at the Protein Data Bank. All other data are contained within the manuscript.

## Supporting information

This article contains supporting information including references (20, 54, 55, 56, 57, 58, 59, 60, 61, 62, 63).

## Conflict of interest

The authors declare that they have no conflicts of interest with the contents of this article.

## References

[1] Marrakchi H., Lanéelle M.-A., Daffé M. (2014). Mycolic acids: structures, biosynthesis, and beyond. Chem. Biol..

[2] Portevin D., De Sousa-D'Auria C., Houssin C., Grimaldi C., Chami M., Daffé M. (2004). A polyketide synthase catalyzes the last condensation step of mycolic acid biosynthesis in mycobacteria and related organisms. Proc. Natl. Acad. Sci. U. S. A..

[3] Gavalda S., Bardou F., Laval F., Bon C., Malaga W., Chalut C. (2014). The polyketide synthase Pks13 catalyzes a novel mechanism of lipid transfer in mycobacteria. Chem. Biol..

[4] Li J., Guddat L.W., Yang H., Zhao Y., Cheng X., Zhang J. (2019). Crystal structures of membrane transporter MmpL3, an anti-TB drug target. Cell.

[5] Daffé M., Marrakchi H. (2019). Unraveling the structure of the mycobacterial envelope. Microbiol Spectr.

[6] Dautin N., de Sousa-d'Auria C., Constantinesco-Becker F., Labarre C., Oberto J., de la Sierra-Gallay I.L. (2017). Mycoloyltransferases: a large and major family of enzymes shaping the cell envelope of *Corynebacteriales*. Biochim. Biophys. Acta..

[7] Brand S., Niehaus K., Pühler A., Kalinowski J. (2003). Identification and functional analysis of six mycolyltransferase genes of *Corynebacterium glutamicum* ATCC 13032: the genes cop1, cmt1, and cmt2 can replace each other in the synthesis of trehalose dicorynomycolate, a component of the mycolic acid layer of the cell envelope. Arch. Microbiol..

[8] De Sousa-D'Auria C., Kacem R., Puech V., Tropis M., Leblon G., Houssin C. (2003). New insights into the biogenesis of the cell envelope of corynebacteria: identification and functional characterization of five new mycoloyltransferase genes in *Corynebacterium glutamicum*. FEMS Microbiol. Lett..

[9] Faller M., Niederweis M., Schulz G.E. (2004). The structure of a mycobacterial outer-membrane channel. Science.

[10] Wang Q., Boshoff H.I.M., Harrison J.R., Ray P.C., Green S.R., Wyatt P.G. (2020). PE/PPE proteins mediate nutrient transport across the outer membrane of *Mycobacterium tuberculosis*. Science.

[11] Niederweis M., Maier E., Lichtinger T., Benz R., Kramer R. (1995). Identification of channel-forming activity in the cell wall of *Corynebacterium glutamicum*. J. Bacteriol..

[12] Lichtinger T., Burkovski A., Niederweis M., Krämer R., Benz R. (1998). Biochemical and biophysical characterization of the cell wall porin of *Corynebacterium glutamicum*: the channel is formed by a low molecular mass polypeptide. Biochemistry.

[13] Ziegler K., Benz R., Schulz G.E. (2008). A putative α-helical porin from *Corynebacterium glutamicum*. J. Mol. Biol..

[14] Huc E., Meniche X., Benz R., Bayan N., Ghazi A., Tropis M. (2010). O-mycoloylated proteins from *Corynebacterium*: an unprecedented post-translational modification in bacteria. J. Biol. Chem..

[15] Huc E., de Sousa-D'Auria C., de la Sierra-Gallay I.L., Salmeron C., van Tilbeurgh H., Bayan N. (2013). Identification of a mycoloyl transferase selectively involved in *O*-acylation of polypeptides in. Corynebacteriales. J. Bacteriol..

[16] Rath P., Demange P., Saurel O., Tropis M., Daffé M., Dötsch V. (2011). Functional expression of the PorAH channel from *Corynebacterium glutamicum* in cell-free expression systems: implications for the role of the naturally occurring mycolic acid modification. J. Biol. Chem..

[17] Issa H., Huc-Claustre E., Reddad T., Bottino N.B., Tropis M., Houssin C. (2017). Click-chemistry approach to study mycoloylated proteins: evidence for PorB and PorC porins mycoloylation in *Corynebacterium glutamicum*. PLoS One.

[18] Carel C., Marcoux J., Réat V., Parra J., Latgé G., Laval F. (2017). Identification of specific posttranslational *O*-mycoloylations mediating protein targeting to the mycomembrane. Proc. Natl. Acad. Sci. U. S. A..

[19] Kacem R., De Sousa-D'Auria C., Tropis M., Chami M., Gounon P., Leblon G. (2004). Importance of mycoloyltransferases on the physiology of *Corynebacterium glutamicum*. Microbiology (N Y).

[20] Migliardo F., Bourdreux Y., Buchotte M., Doisneau G., Beau J.-M., Bayan N. (2019). Study of the conformational behaviour of trehalose mycolates by FT-IR spectroscopy. Chem. Phys. Lipids..

[21] Rath P., Saurel O., Czaplicki G., Tropis M., Daffé M., Ghazi A. (2013). Cord factor (trehalose 6,6′-dimycolate) forms fully stable and non-permeable lipid bilayers required for a functional outer membrane. Biochim. Biophys. Acta Biomembr..

[22] Lesur E., Rollando P., Guianvarc'h D., Bourdreux Y. (2023). Synthesis of trehalose-based chemical tools for the study of the mycobacterial membrane. Comptes rendus. Chimie. Online first.

[23] Lesur E., Baron A., Dietrich C., Buchotte M., Doisneau G., Urban D. (2019). First access to a mycolic acid-based bioorthogonal reporter for the study of the mycomembrane and mycoloyltransferases in Corynebacteria. Chem. Commun..

[24] Labarre C., Zhang Y., Lesur E., Ley M., Sago L., Dietrich C. (2024). Bioorthogonal monomycolate of trehalose disclosed the *O*-mycoloylation of mycoloyltransferases and other cell envelope proteins in *C. glutamicum*. ACS Chem. Biol..

[25] Noyori R., Ohkuma T., Kitamura M., Takaya H., Sayo N., Kumobayashi H. (1987). Asymmetric hydrogenation of β-keto carboxylic esters. A practical, purely chemical access to β-hydroxy esters in high enantiomeric purity. J. Am. Chem. Soc..

[26] Fráter G. (1979). Über die Stereospezifität der α-Alkylierung von β-Hydroxycarbonsäureestern. Vorläufige Mitteilung. Helv. Chim. Acta.

[27] Seebach D., Wasmuth D. (1980). Herstellung von erythro-2-Hydroxybernsteinsäure-Derivaten aus Äpfelsäureester. Vorläufige Mitteilung. Helv. Chim. Acta.

[28] Belisle J.T., Vissa V.D., Sievert T., Takayama K., Brennan P.J., Besra G.S. (1997). Role of the major antigen of *Mycobacterium tuberculosis* in cell wall biogenesis. Science.

[29] Boucau J., Sanki A.K., Voss B.J., Sucheck S.J., Ronning D.R. (2009). A coupled assay measuring Mycobacterium tuberculosis antigen 85C enzymatic activity. Anal. Biochem..

[30] Favrot L., Grzegorzewicz A.E., Lajiness D.H., Marvin R.K., Boucau J., Isailovic D. (2013). Mechanism of inhibition of Mycobacterium tuberculosis antigen 85 by ebselen. Nat. Commun..

[31] Ronning D.R., Klabunde T., Besra G.S., Vissa V.D., Belisle J.T., Sacchettini J.C. (2000). Crystal structure of the secreted form of antigen 85C reveals potential targets for mycobacterial drugs and vaccines. Nat. Struct. Biol..

[32] Ronning D.R., Vissa V., Besra G.S., Belisle J.T., Sacchettini J.C. (2004). Mycobacterium tuberculosis antigen 85A and 85C structures confirm binding orientation and conserved substrate specificity. J. Biol. Chem..

[33] Richard M., Blaise M., Cavalier J.-F., Viljoen A., Paudal R.R., Gnawali G.R. (2018). Cyclipostins and cyclophostin analogs inhibit the antigen 85C from Mycobacterium tuberculosis both *in vitro* and *in vivo*. J. Biol. Chem..

[34] Goins C.M., Dajnowicz S., Smith M.D., Parks J.M., Ronning D.R. (2018). Mycolyltransferase from Mycobacterium tuberculosis in covalent complex with tetrahydrolipstatin provides insights into antigen 85 catalysis. J. Biol. Chem..

[35] Klatt S., Brammananth R., O'Callaghan S., Kouremenos K.A., Tull D., Crellin P.K. (2018). Identification of novel lipid modifications and intermembrane dynamics in Corynebacterium glutamicum using high-resolution mass spectrometry. J. Lipid Res..

[36] Wang H.Y.J., Tatituri R.V.V., Goldner N.K., Dantas G., Hsu F.F. (2020). Unveiling the biodiversity of lipid species in Corynebacteria- characterization of the uncommon lipid families in C. glutamicum and pathogen C. striatum by mass spectrometry. Biochimie.

[37] Hodges H.L., Brown R.A., Crooks J.A., Weibel D.B., Kiessling L.L. (2018). Imaging mycobacterial growth and division with a fluorogenic probe. Proc. Natl. Acad. Sci. U. S. A.

[38] Kavunja H.W., Piligian B.F., Fiolek T.J., Foley H.N., Nathan T.O., Swarts B.M. (2016). A chemical reporter strategy for detecting and identifying O-mycoloylated proteins in Corynebacterium. Chem. Commun. (Camb).

[39] Backus K.M., Dolan M.A., Barry C.S., Joe M., McPhie P., Boshoff H.I.M. (2014). The three Mycobacterium tuberculosis antigen 85 isoforms have unique substrates and activities determined by non-active site regions. J. Biol. Chem..

[40] Asselineau C., Tocanne G., Tocanne J.F. (1970). Stereochemistry of mycolic acids. Bull. Soc. Chim. Fr.

[41] Goins C.M., Schreidah C.M., Dajnowicz S., Ronning D.R. (2018). Structural basis for lipid binding and mechanism of the Mycobacterium tuberculosis Rv3802 phospholipase. J. Biol. Chem..

[42] Dietrich C., Li de la Sierra-Gallay I., Masi M., Girard E., Dautin N., Constantinesco-Becker F. (2020). The C-terminal domain of Corynebacterium glutamicum mycoloyltransferase A is composed of five repeated motifs involved in cell wall binding and stability. Mol. Microbiol..

[43] Schägger H. (2006). Tricine-SDS-PAGE. Nat. Protoc..

[44] Bou Raad R., Meniche X., de Sousa-d'Auria C., Chami M., Salmeron C., Tropis M. (2010). A deficiency in arabinogalactan biosynthesis affects Corynebacterium glutamicum mycolate outer membrane stability. J. Bacteriol..

[45] Kabsch W. (2010). XDS. Acta Crystallogr. D Biol. Crystallogr..

[46] Vagin A., Teplyakov A. (2010). Molecular replacement with MOLREP. Acta Crystallogr. D Biol. Crystallogr..

[47] Agirre J., Atanasova M., Bagdonas H., Ballard C.B., Baslé A., Beilsten-Edmands J. (2023). The CCP4 suite: integrative software for macromolecular crystallography. Acta Crystallogr. D Struct. Biol..

[48] Emsley P., Cowtan K. (2004). Coot: model-building tools for molecular graphics. Acta Crystallogr. D Biol. Crystallogr..

[49] Bricogne G., Blanc E., Brandl M., Flensbueg C., Keller P., Paciorek W. (2011).

[50] Liebschner D., Afonine P.V., Moriarty N.W., Poon B.K., Sobolev O.V., Terwilliger T.C. (2017). Polder maps: improving OMIT maps by excluding bulk solvent. Acta Crystallogr. D Struct. Biol..

[51] Mirdita M., Schütze K., Moriwaki Y., Heo L., Ovchinnikov S., Steinegger M. (2022). ColabFold: making protein folding accessible to all. Nat. Methods.

[52] Evans R., O'Neill M., Pritzel A., Antropova N., Senior A., Green T. (2022). Protein complex prediction with AlphaFold-Multimer. bioRxiv.

[53] Jumper J., Evans R., Pritzel A., Green T., Figurnov M., Ronneberger O. (2021). Highly accurate protein structure prediction with AlphaFold. Nature.

[54] Van Der Peet P.L., Gunawan C., Torigoe S., Yamasaki S., Williams S.J. (2015). Corynomycolic acid-containing glycolipids signal through the pattern recognition receptor Mincle. Chem. Commun..

[55] Ratovelomanana-Vidal V., Girard C., Touati R., Tranchier J.P., Ben Hassine B., Genêt J.P. (2003). Enantioselective hydrogenation of β-keto esters using chiral diphosphine-ruthenium complexes: optimization for academic and industrial purposes and synthetic applications. Adv. Synth. Catal..

[56] Yamamoto H., Oda M., Nakano M., Watanabe N., Yabiku K., Shibutani M. (2013). Development of vizantin, a safe immunostimulant, based on the structure-activity relationship of trehalose-6,6′-dicorynomycolate. J. Med. Chem..

[57] Brooks D.W., Lu L.D.-L., Masamune S. (1979). C-Acylation under virtually neutral conditions. Angew. Chem. Int. Edition English.

[58] Seifert T., Malo M., Kokkola T., Stéen E.J.L., Meinander K., Wallén E.A.A. (2020). A scaffold replacement approach towards new sirtuin 2 inhibitors. Bioorg. Med. Chem..

[59] Muto S.E., Mori K. (2003). Synthesis of the four components of the female sex pheromone of the painted apple moth, teia anartoides. Biosci. Biotechnol. Biochem..

[60] Genêt J.P., Pinel C., Ratovelomanana-Vidal V., Mallart S., Pfister X., De Andrade M.C.C. (1994). Novel, general synthesis of the chiral catalysts diphosphine-ruthenium (II) diallyl complexes and a new practical in situ preparation of chiral ruthenium (II) catalysts. Tetrahedron Asymmetry.

[61] Radivojevic J., Skaro S., Senerovic L., Vasiljevic B., Guzik M., Kenny S.T. (2016). Polyhydroxyalkanoate-based 3-hydroxyoctanoic acid and its derivatives as a platform of bioactive compounds. Appl. Microbiol. Biotechnol..

[62] Toubiana R., Das B.C., Defaye J., Mompon B., Toubiana M.-J. (1975). Étude du cord-factor et de ses analogues. Carbohydr. Res..

[63] Kallerup R.S., Franzyk H., Schiøth M.L., Justesen S., Martin-Bertelsen B., Rose F. (2017). Adjuvants based on synthetic mycobacterial cord factor analogues: biophysical properties of neat glycolipids and nanoself-assemblies with DDA. Mol. Pharm..

