# Supplementary material for: Synthetic mycolates derivatives to decipher protein mycoloylation, a unique post-translational modification in bacteria
Source: J Biol Chem. 2025 Jan 27;301(3):108243. doi: 10.1016/j.jbc.2025.108243 (PMC11927696; doi:10.1016/j.jbc.2025.108243)
Supplement: Supporting information [file mmc1.docx]

**Supporting Information for**

**Synthetic mycolates derivatives to decipher protein mycoloylation, a unique post-translational modification in bacteria**.

Emilie Lesur *^1^, Yijie Zhang*^2^, Nathalie Dautin^2, 3^, Christiane Dietrich^2^, Ines Li de la Sierra-Gallay^2^, Luis Augusto^2^, Paulin Rollando^1^, Noureddine Lazar^2^, Dominique Urban^1^, Gilles Doisneau^1^, Florence Constantinesco-Becker^2^, Herman Van Tilbeurgh^2$^, Dominique Guianvarc’h^1$^, Yann Bourdreux^1^ and Nicolas Bayan^2$^

Affiliations:

^1^ Université Paris-Saclay, CNRS, Institut de Chimie Moléculaire et des Matériaux d'Orsay (ICMMO), UMR 8182, F-91405 Orsay, France

^2^ Université Paris-Saclay, CEA, CNRS, Institute for Integrative Biology of the Cell (I2BC), 91198, Gif-sur-Yvette, France.

^3^ present address: Université Paris Cité, CNRS, Biochimie des Protéines Membranaires, F-75005 Paris, France.

* Both authors equally contributed to this work

^$^ Correspondance to [nicolas.bayan@i2bc.paris-saclay.fr](mailto:nicolas.bayan@i2bc.paris-saclay.fr) or [dominique.guianvarch@universite-paris-saclay.fr](mailto:dominique.guianvarch@universite-paris-saclay.fr) or [herman.van-tilbeurgh@i2bc.paris-saclay.fr](mailto:herman.van-tilbeurgh@i2bc.paris-saclay.fr)

Running Title: Synthetic mycolates derivatives and protein mycoloylation.

Keywords: membrane, lipid metabolism, post translational modification, mycoloyltransferase, protein lipidation, Mycobacteria.

This document contains:

- Supporting methods for chemical synthesis
- Figures S1 to S7
- SI References

Supporting methods for chemical synthesis

**General methods:**

All air sensitive reactions were carried out in oven-dried glassware under a slight positive pressure of argon. Solvents were dried by standard methods. THF was distilled from sodium benzophenone ketyl. TLC (Silica Gel 60 F_254_) were visualized under UV (254 nm) and by staining either in 5% ethanolic sulfuric acid or orcinol or phosphomolybdic acid. Silica gel SDS 60 ACC 35-70 μm was used for column chromatography. NMR spectra were recorded on Bruker DRX 300 or AV 360 spectrometers. Chemical shifts (in ppm) were determined relative to residual undeuterated solvent as an internal reference. Abbreviations of multiplicity were as follows: s (singlet), d (doublet), dd (doublet of doublet), t (triplet), at (apparent triplet), m (multiplet), b (broad). Coupling constants in hertz (Hz) were measured from one-dimensional spectra. High-resolution mass spectra (positive or negative mode ESI) were performed on a Bruker Daltonics micrOTOF-QII spectrometer. Optical rotations were measured on an Anton Paar MCP 150 polarimeter (*c* in g / 100 mL). Synthetic TMM, TDM, **9b** and **10b** were synthesized according to the literature (20, 54, 55).

**Tetradecyl-octadecanoic acid 8**

To a solution of dry diisopropylamine (830 µL, 5.92 mmol, 2.5 equiv) in THF (5 mL) cooled to 0 °C was added dropwise *n*-butyllithium (3.5 mL, 1.6 M, 5.60 mmol, 2.4 equiv). After stirring for 30 min at 0 °C the solution was added, *via* cannula, to a solution of commercially available palmitic acid (600 mg, 2.34 mmol) in dry THF (10 mL). The reaction mixture was stirred for 1 h at 0 °C and then, iodohexadecane (800 µL, 2.76 mmol, 1.2 equiv) and HMPA (500 µL, 2.87 mmol, 1.2 equiv) were added slowly. The mixture was warmed to 50 °C and stirred overnight at this temperature. The mixture was acidified with an aqueous HCl solution (1M, pH ≈ 1) and extracted three times with EtOAc. The combined organic layers were washed with an aqueous NaCl solution, dried over Na_2_SO_4_ and concentrated under reduced pressure. The residue was purified by flash silica gel chromatography (cyclohexane/EtOAc, 13:1 to 12:1) to give compound **8** (515 mg, 46%) as a colourless oil. NMR data were in agreement with literature (56). R_f_ **=** 0.45 (cyclohexane/EtOAc, 13:1). ^1^H NMR (CDCl_3_, 360 MHz) δ (ppm): 2.35 (m, 1H, H_b_), 1.70-1.39 (m, 4H, H_c_ and H_a’_), 1.40-1.12 (m, 52H, H_c_-H_o_ and H_b’_-H_o’_), 0.90 (t, 6H, *J* = 7.0 Hz, H_p’_ and H_p_). ^13^C NMR (CDCl_3_, 90 MHz) δ (ppm): 180.2 (C_a_), 45.4 (C_b_), 32.1, 31.9, 29.6, 29.5, 29.4, 29.3, 27.3, 22.6 (28C, C_c_-C_o_, C_a’_-C_o’_), 14.1 (2C, C_p_, C_p’_). HRMS (ESI): calcd for C_32_H_63_O_2_ [M-H]^-^ 479.4834, found 479.4812.

**Methyl 3-oxo-octanoate 9a:**

Compound **9a** was obtained according to Masamune’s procedure (57). Briefly, carbonyldiimidazole (1.68 g, 10.32 mmol, 1.2 equiv) was added to a solution of commercially available hexanoic acid (1.1 mL, 8.80 mmol) in dry THF (84 mL). After stirring at room temperature for 6-8 h, the magnesium salt of monomethylmalonate (2.89 g, 11.22 mmol, 1.3 equiv) was added. The reaction mixture was stirred for 18 h at room temperature and was then acidified with an aqueous HCl solution (1M, pH ≈ 1). The reaction mixture was extracted three times with EtOAc, and the combined organic layers were dried over Na_2_SO_4_ and concentrated under reduced pressure. The residue was purified by flash silica gel chromatography (cyclohexane to cyclohexane/EtOAc 99:1) to give compound **9a** (869 mg, 59%) as a colourless oil. Spectral data were in agreement with literature (58). R_f_ **=** 0.49 (cyclohexane/EtOAc, 85:15). ^1^H NMR (CDCl_3_, 300 MHz) δ (ppm): 3.74 (s, 3H, OCH_3_), 3.44 (s, 2H, H_b_), 2.52 (t, 2H, *J* = 7.4 Hz, H_d_), 1.66-1.52 (m, 2H, H_e_), 1.34-1.22 (m, 4H, H_f_, H_g_), 0.89 (t, 3H, *J* = 6.9 Hz, H_h_). ^13^C NMR (CDCl_3_, 75 MHz) δ (ppm): 203.0 (C_c_), 167.8 (C_a_), 52.5 (OCH_3_), 49.2 (C_b_), 43.2 (C_d_), 31.3 (C_f_), 23.3 (C_e_), 22.5 (C_g_), 14.0 (C_h_). HRMS (ESI): calcd for C_9_H_16_NaO_3_ [M+Na]^+^ 195.0992, found 195.0983, calcd for C_9_H_17_O_3_ [M+H]^+^ 173.1172, found 173.1166.

**Methyl 3-oxo-tridecanoate 9c**

Compound **9c** was synthetized according to Masamune’s procedure (57). Carbonyldiimidazole (1.04 g, 6.44 mmol, 1.2 equiv) was added to a solution of commercially available undecanoic acid (1 g, 5.37 mmol) in dry THF (55 mL). After stirring at room temperature for 6-8 h, the magnesium salt of monomethylmalonate (1.80 g, 6.98 mmol, 1.3 equiv) was added. The reaction mixture was stirred for 18 h at room temperature and was then acidified with an aqueous HCl solution (1M, pH ≈ 1). The reaction mixture was extracted three times with EtOAc, and the combined organic layers were dried over Na_2_SO_4_ and concentrated under reduced pressure. The residue was purified by flash silica gel chromatography (cyclohexane/EtOAc, 98:2 to 95:5) to give compound **9c** (679 mg, 52%) as a colourless oil. NMR data were in agreement with literature (59). R_f_ **=** 0.50 (cyclohexane/EtOAc, 9:1). ^1^H NMR (CDCl_3_, 300 MHz) δ (ppm): 3.74 (s, 3H, OCH_3_), 3.45 (s, 2H, H_b_), 2.52 (t, 2H, *J =* 7.5 Hz, H_d_), 1.65-1.54 (m, 2H, H_e_), 1.34-1.20 (m, 14H, H_f_-H_l_), 0.87 (t, 3H, H_m_). ^13^C NMR (CDCl_3_, 75 MHz) δ (ppm): 203.1 (C_c_), 167.9 (C_a_), 52.5 (OCH_3_), 49.2 (C_b_), 43.2 (C_d_), 32.0, 29.7, 29.6, 29.5, 29.4, 29.1, 22.8 (7C, C_f_-C_l_), 19.8 (C_k_), 23.6 (C_e_), 14.3 (C_m_). HRMS (ESI): calcd for C_14_H_26_NaO_3_ [M+Na]^+^ 265.1774, found 265.1766, calcd for C_14_H_27_O_3_ [M+H]^+^ 243.1955, found 243.1943.

**Methyl (*R*)-3-hydroxy-octanoate 10a**

The (*R*)-BINAP-RuBr_2_ complex was prepared under argon according to a reported procedure (60). To a solution of (*R*)-BINAP (77 mg, 0.12 mmol, 0.022 equiv) and (Cod)Ru(2-methylallyl)_2_ (36 mg, 0.11 mmol, 0.02 equiv) in anhydrous and degassed acetone (6 mL), were added 1.5 mL of a methanolic HBr solution (0.165 M, 0.25 mmol, 0.044 equiv). The reaction mixture was stirred for 30 min at room temperature and then concentrated under vacuum. A solution of β-ketoester **9a** (970 mg, 5.6 mmol) in degassed and dry MeOH (6.9 mL) was added *via* cannula to the catalyst. The mixture was purged three times with dihydrogen and vigorously stirred overnight at 50 °C under 1 atm of dihydrogen. The solvent was evaporated under vacuum and the residue was purified by silica gel chromatography (cyclohexane/EtOAc, 99:1 to 95:5) to give the desired product **10a** (878 mg, 90%) as a colourless oil. NMR data were in agreement with literature (61). R_f_ **=** 0.23 (cyclohexane/EtOAc, 85:15). ^1^H NMR (CDCl_3_, 360 MHz) δ (ppm): 4.00 (m, 1H, H_c_), 3.71 (s, 3H, OCH_3_), 2.52 (dd, 1H, *J* = 16.4 Hz, *J* = 3.2 Hz, H_b_), 2.41 (dd, 1H, *J* = 16.4 Hz, *J* = 8.7 Hz, H_b’_), 1.59-1.23 (m, 8H, H_d_-H_g_), 0.89 (t, 3H, *J* = 6.5 Hz, H_h_). ^13^C NMR (CDCl_3_, 90 MHz) δ (ppm): 173.4 (C_a_), 68.0 (C_c_), 51.7 (OCH_3_), 41.3 (C_b_), 36.6, 31.7, 25.2, 22.6 (4C, C_d_-C_g_), 14.0 (C_h_). HRMS (ESI): calcd for C_9_H_18_NaO_3_ [M+Na]^+^ 197.1148, found 197.1143. ${[\alpha]}_{D}^{20}$ **=** -19 (*c* 1.07, CHCl_3_).

**Methyl (*R*)-3-hydroxy-tridecanoate 10c**

The (*R*)-BINAP-RuBr_2_ complex was prepared under argon according to a reported procedure (60). To a solution of (*R*)-BINAP (41.9 mg, 0.067 mmol, 0.022 equiv) and (Cod)Ru(2-methylallyl)_2_ (19.5 mg, 0.061 mmol, 0.020 equiv) in anhydrous and degassed acetone, were added 820 µL of a methanolic HBr solution (0.165 M, 0.135 mmol, 0.044 equiv). The reaction mixture was stirred for 30 min at room temperature and then concentrated under vacuum. A solution of β-ketoester **9c** (742 mg, 3.06 mmol) in degassed and dry MeOH (3.75 mL) was added *via* cannula to the catalyst. The mixture was purged three times with dihydrogen and vigorously stirred overnight at 50 °C under 1 atm of dihydrogen. The solvent was evaporated under vacuum and the residue was purified by silica gel chromatography (cyclohexane/EtOAc, 95:5 to 90:10) to give the desired product **10c** (570.1 mg, 76%) as a colourless oil. R_f_ **=** 0.21 (cyclohexane/EtOAc, 9:1). ^1^H NMR (CDCl_3_, 360 MHz) δ (ppm): 3.97 (m, 1H, H_c_), 3.69 (s, 3H, OCH_3_), 2.95 (s, 1H, OH), 2.49 (dd, 1H, *J =* 16.3 Hz, *J =* 3.2 Hz, H_b_), 2.38 (dd, 1H, *J =* 16.3 Hz, *J* = 8.9 Hz, H_b’_), 1.54-1.16 (m, 18H, H_d_-H_l_), 0.85 (t, 3H, H_m_). ^13^C NMR (CDCl_3_, 90 MHz) δ (ppm): 173.6 (C_a_), 68.1 (C_c_), 51.8 (OCH_3_), 41.2 (C_b_), 36.6 (C_d_), 32.0, 29.7, 29.6, 29.4, 25.6, 22.8 (9C, C_d_-C_l_), 14.2 (C_m_). HRMS (ESI): calcd for C_14_H_28_NaO_3_ [M+Na]^+^ 267.1931, found 267.1924. ${[\alpha]}_{D}^{20}$ **=** -14 (*c* 1.13, CHCl_3_).

**(*R*)-3-triethylsilyloxy-octadecanoic acid 11**

A 10% aqueous solution of NaOH (16 mL) was added to a solution of compound **10b** (55) (501.9 mg, 1.60 mmol) in MeOH (16 mL). The reaction mixture was stirred overnight at room temperature and then acidified to pH ≈ 1 with an aqueous HCl solution (1M). The mixture was extracted with EtOAc, the combined organic layers were dried over Na_2_SO_4_ and concentrated under reduced pressure. The crude product was purified by flash silica gel chromatography (EtOAc/MeOH, 99:1 to 97:3) to give the corresponding carboxylic acid (426.3 mg, 89%) as a colourless syrup. NMR data were in agreement with literature (55). R_f_ **=** 0.1 (cyclohexane/EtOAc, 1:1). ^1^H NMR (CDCl_3_, 360 MHz) δ (ppm): 4.04 (m, 1H, H_c_), 2.60 (dd, 1H, *J =* 16.7 Hz, *J =* 3.2 Hz, H_b_), 2.49 (dd, 1H, *J =* 16.5 Hz, *J* = 8.9 Hz, H_b’_), 1.65-1.15 (m, 28H, H_d_-H_q_), 0.89 (t, 3H, J = 7.0 Hz, H_r_). ^13^C NMR (CDCl_3_, 75 MHz) δ (ppm): 175.0 (C_a_), 68.0 (C_c_), 36.5 (C_b_), 31.8, 29.5, 29.2, 25.4, 22.5 (14C, C_d_-C_q_), 13.8 (C_r_). HRMS (ESI): calcd for C_18_H_36_NaO_3_ [M+Na]^+^ 323.2557, found 323.2551. ${[\alpha]}_{D}^{20}$ **=** -1 (*c* 1.0, CHCl_3_/MeOH 1/1). To a solution of this compound (92 mg, 0.3 mmol) in dry pyridine (2 mL) was added dropwise TESCl (115 µL, 0.67 mmol, 2.2 equiv). The reaction mixture was stirred overnight at room temperature and was then diluted with a saturated aqueous NaCl s olution. The reaction mixture was extracted three times with EtOAc. The combined organic layers were dried over Na_2_SO_4_ and concentrated under reduced pressure. The residue was purified by flash silica gel chromatography (cyclohexane/EtOAc, 80:20) to give compound **11** (75 mg, 59%) as a colourless oil. R_f_ **=** 0.60 (cyclohexane/EtOAc 8:2). ^1^H NMR (CDCl_3_, 360 MHz) δ (ppm): 4.05 (m, 1H, H_c_), 2.60 (dd, 1H, *J* = 15.4 Hz, *J* = 5.0 Hz, H_b_), 2.50 (dd, 1H, *J* = 15.4 Hz, *J* = 4.5 Hz, H_b’_), 1.62-1.46 (m, 2H, H_d_), 1.33-1.19 (m, 26H, H_e_-H_q_), 0.98 (t, 9H, *J* = 7.6 Hz, Si(CH_2_C*H*_3_)_3_), 0.88 (t, 3H, *J* = 7.0 Hz, H_r_), 0.66 (q, 6H, *J* = 7.6 Hz, Si(C*H*_2_CH_3_)_3_). ^13^C NMR (CDCl_3_, 90 MHz) δ (ppm): 174.6 (C_a_), 69.6 (C_c_), 41.6 (C_b_), 37.3 (C_d_), 32.0, 29.8, 29.7, 29.5, 25.5, 22.9 (13C, C_e_-C_q_), 14.3 (C_r_), 6.9 (Si(CH_2_*C*H_3_)_3_), 5.0 (Si(*C*H_2_CH_3_)_3_). HRMS (ESI): calcd for C_24_H_50_NaO_3_Si [M+Na]^+^ 437.3421, found 437.3400. ${[\alpha]}_{D}^{20}$ **=** - 1 (*c* 0.96, CHCl_3_).

**Methyl (2*R*, 3*R*)-2-pentyl-3-hydroxy-octanoate 13a**

To a solution of dry diisopropylamine (2 mL, 14.27 mmol, 2.8 equiv) in THF (13.6 mL) cooled to -40 °C were added dropwise *n*-butyllithium (8 mL, 1.6 M, 12.8 mmol, 2.5 equiv). After stirring for 45 min at -40 °C, the mixture was cooled to -78 °C and a solution of β-hydroxyester **10a** (878 mg, 5.04 mmol) in dry THF (29 mL) was added *via* cannula. The reaction mixture was stirred for 1h30 at -78 °C then iodopentane (2 mL, 15.12 mmol, 3 equiv) and HMPA (1.05 mL, 6.05 mmol, 1.2 equiv) were added slowly. The mixture was warmed slowly to -10 °C and stirred overnight at this temperature. The mixture was diluted with a saturated aqueous NH_4_Cl solution and extracted three times with EtOAc. The combined organic layers were washed with an aqueous NaCl solution, dried over Na_2_SO_4_ and concentrated under reduced pressure. The residue was purified by flash silica gel chromatography (cyclohexane/EtOAc, 95:5) to give compound **13a** (357.3 mg, 29%) as a colourless oil. R_f_ **=** 0.46 (cyclohexane/EtOAc, 85:15). ^1^H NMR (CDCl_3_, 360 MHz) δ (ppm): 3.70 (s, 3H, OCH_3_), 3.65 (m, 1H, H_c_), 2.48-2.39 (m, 2H, OH and H_b_), 1.69 (m, 1H, H_a’_), 1.66 (m, 1H, H_a’_), 1.50-1.20 (m, 14H, H_d_-H_g_, H_b’_-H_d’_), 0.88 (t, 3H, *J* = 6.6 Hz, H_h_ or H_e’_), 0.87 (t, 3H, *J* = 6.5 Hz, H_e’_ or H_h_). ^13^C NMR (CDCl_3_, 90 MHz) δ (ppm): 176.4 (C_a_), 72.4 (C_c_), 51.7 (OCH_3_), 51.1 (C_b_), 35.8, 31.9, 31.8, 29.7, 27.2, 25.5, 22.7, 22.6 (8C, C_d_-C_g_, C_a’_-C_d’_), 14.2, 14.1 (2C, C_h_, C_e’_). HRMS (ESI): calcd for C_14_H_28_NaO_3_ [M+Na]^+^ 267.1931, found 267.1922. ${[\alpha]}_{D}^{20}$ **=** + 12 (*c* 0.77, CHCl_3_).

**(2*R*, 3*R*)-2-pentyl-3-triethylsilyloxy-octanoic acid 14a**

A 10% aqueous NaOH solution (2.9 mL) was added to compound **13a** (95 mg, 0.39 mmol) in MeOH (2.9 mL). The reaction mixture was stirred overnight at 45 °C and then acidified to pH ≈ 1 with an aqueous HCl solution (1M). The mixture was extracted three times with EtOAc, the combined organic layers were dried over Na_2_SO_4_ and concentrated under reduced pressure. The residue was purified by flash silica gel chromatography (cyclohexane/EtOAc, 80:20) to give the corresponding carboxylic acid (65 mg, 73%) as a colourless oil. R_f_ **=** 0.33 (cyclohexane/EtOAc, 1:1). ^1^H NMR (CDCl_3_, 360 MHz) δ (ppm): 3.72 (m, 1H, H_c_), 2.47 (td, 1H, *J* = 9.0 Hz, *J* = 5.3 Hz, H_b_), 1.75 (m, 1H, H_a’_), 1.62 (m, 1H, H_a’_), 1.55-1.23 (m, 14H, H_d_-H_g_, H_b’_-H_d’_), 0.89 (t, 3H, *J* = 6.7 Hz, H_h_ or H_e’_), 0.88 (t, 6H, *J* = 6.7 Hz, H_e’_ or H_h_). ^13^C NMR (CDCl_3_, 90 MHz) δ (ppm): 180.7 (C_a_), 72.3 (C_c_), 51.1 (C_b_), 35.6, 31.8, 29.6, 27.1, 25.5, 22.7, 22.6 (8C, C_d_-C_g_, C_a’_-C_d’_), 14.2, 14.1 (2C, C_h_, C_e’_). HRMS (ESI): calcd for C_33_H_26_NaO_3_ [M+Na]^+^ 253.1774, found 253.1764. ${[\alpha]}_{D}^{20}$ **=** +15 (*c* 1.09, CHCl_3_). To a solution of this compound (52 mg, 0.23 mmol) in dry pyridine (2 mL) was added dropwise TESCl (190 µL, 1.13 mmol, 5 equiv). The reaction mixture was stirred overnight at 60 °C and was then diluted with a saturated aqueous NaCl solution. The reaction mixture was extracted three times with EtOAc. The combined organic layers were dried over Na_2_SO_4_ and concentrated under reduced pressure. The residue was purified by flash silica gel chromatography (cyclohexane/EtOAc, 98:2) to give compound **14a** (29.6 mg, 38%) as a colourless oil. R_f_ **=** 0.63 (cyclohexane/EtOAc 85:15). ^1^H NMR (CDCl_3_, 360 MHz) δ (ppm): 3.87 (m, 1H, H_c_), 2.51 (m, 1H, H_b_), 1.71-1.59 (m, 1H, H_a’_), 1.58-1.47 (m, 3H, H_d_, H_a’_), 1.40-1.22 (m, 12H, H_e_-H_g_, H_b’_-H_d’_), 0.97 (t, 9H, *J* = 7.9 Hz, Si(CH_2_C*H*_3_)_3_), 0.88 (t, 6H, *J* = 6.9 Hz, H_h_, H_e’_), 0.64 (q, 6H, *J* = 7.9 Hz, Si(C*H*_2_CH_3_)_3_). ^13^C NMR (CDCl_3_, 90 MHz) δ (ppm): 177.6 (C_a_), 73.8 (C_c_), 51.0 (C_b_), 35.4, 32.0, 31.8, 29.2, 27.4, 24.7, 22.7, 22.6 (8C, C_d_-C_g_, C_a’_-C_d’_), 14.1 (2C, C_h_, C_e’_), 6.9 (Si(CH_2_*C*H_3_)_3_), 5.1 (Si(*C*H_2_CH_3_)_3_). HRMS (ESI): calcd for C_19_H_41_O_3_Si [M+H]^+^ 345.2819, found 345.2802, calcd for C_21_H_40_NaO_3_Si [M+Na]^+^ 367.2639, found 367.2621. ${[\alpha]}_{D}^{20}$ **=** + 7 (*c* 1.5, CHCl_3_).

**Methyl (2*R*, 3*R*)-2-decyl-3-hydroxy-tridecanoate 13b**

To a solution of dry diisopropylamine (722 µL, 7.16 mmol, 3.5 equiv) in THF (5.9 mL) cooled to -40 °C were added dropwise 3.84 mL of *n*-butyllithium (6.14 mmol, 1.6 M in hexane, 3 equiv). After stirring for 40 min at -40 °C, the mixture was cooled to -78 °C and a solution of β-hydroxyester **10c** (500 mg, 2.05 mmol) in dry THF (14.9 mL) was added *via* cannula. The reaction mixture was stirred for 1h30 at -78 °C then 1-iododecane (1.31 mL, 6.14 mmol, 3 equiv) and HMPA (427 µL, 2.46 mmol, 1.2 equiv) were added slowly. The mixture was warmed slowly to -10 °C and stirred overnight at this temperature. The mixture was diluted with a saturated aqueous NH_4_Cl solution and extracted three times with EtOAc. The combined organic layers were washed with an aqueous NaCl solution, dried over Na_2_SO_4_ and concentrated under reduced pressure. The residue was purified by flash silica gel chromatography (cyclohexane/EtOAc, 98:2 to 95:5) to give compound **13b** (185 mg, 27%) as a colourless oil. R_f_ **=** 0.47 (cyclohexane/EtOAc, 85:15). ^1^H NMR (CDCl_3_, 300 MHz) δ (ppm): 3.70 (s, 3H, OCH_3_), 3.65 (m, 1H, H_c_), 2.43 (m, 1H, H_b_), 2.35 (bs, 1H, OH), 1.79-1.51 (m, 2H, H_a’_), 1.51-1.15 (m, 34H, H_d_-H_l_, H_b’_-H_i’_), 0.87 (t, 6H, *J* = 6.7 Hz, H_m_, H_j’_). ^13^C NMR (CDCl_3_, 75 MHz) δ (ppm): 176.4 (C_a_), 72.4 (C_c_), 51.7 (OCH_3_), 51.1 (C_b_), 35.8, 32.0, 29.8, 29.7, 29.6, 29.5, 29.4, 27.6, 25.6, 22.8 (18C, C_d_-C_l_, C_a’_-C_i’_), 14.3 (2C, C_m_, C_j’_). HRMS (ESI): calcd for C_24_H_48_NaO_3_ [M+Na]^+^ 407.3496, found 407.3478. ${[\alpha]}_{D}^{20}$ **=** + 9 (*c* 1.02, CHCl_3_).

**(2*R*, 3*R*)-2-Decyl-3-triethylsilyloxy-tridecanoic acid 14b**

A 10% aqueous NaOH solution (6 mL) was added to compound **13b** (185 mg, 0.48 mmol) in MeOH (6 mL). The reaction mixture was stirred overnight at 45 °C and then acidified to pH ≈ 1 with an aqueous HCl solution (1M). The mixture was extracted three times with EtOAc, the combined organic layers were dried over Na_2_SO_4_ and concentrated under reduced pressure. The residue was purified by flash silica gel chromatography (cyclohexane/EtOAc, 98:2 to 95:5) to give the corresponding carboxylic acid (143.9 mg, 80%) as a colourless oil. R_f_ **=** 0.05 (cyclohexane/EtOAc, 8:2). ^1^H NMR (CDCl_3_, 300 MHz) δ (ppm): 3.71 (m, 1H, H_c_), 2.45 (td, 1H, *J* = 8.8 Hz, *J* = 5.4 Hz, H_b_), 1.79-1.19 (m, 36H, H_d_-H_l_, H_a’_-H_i’_), 0.88 (t, 6H, *J* = 6.8 Hz, H_m_, H_j’_). ^13^C NMR (CDCl_3_, 75 MHz) δ (ppm): 180.3 (C_a_), 72.3 (C_c_), 51.1 (C_b_), 35.6, 32.1, 29.8, 29.7, 29.6, 29.5, 27.4, 25.9, 22.8 (18C, C_d_-C_l_, C_a’_-C_i’_), 14.3 (2C, C_m_, C_j’_). HRMS (ESI): calcd for C_23_H_46_NaO_3_ [M+Na]^+^ 393.3339, found 393.3323. ${[\alpha]}_{D}^{20}$ **=** +12 (*c* 0.99, CHCl_3_). To a solution of this compound (100 mg, 0.27 mmol) in dry pyridine (2.4 mL) was added dropwise TESCl (226 µL, 1.35 mmol, 5 equiv). The reaction mixture was stirred overnight at 60 °C and was then diluted with a saturated aqueous NaCl solution. The reaction mixture was extracted three times with EtOAc. The combined organic layers were dried over Na_2_SO_4_ and concentrated under reduced pressure. The residue was purified by flash silica gel chromatography (cyclohexane/EtOAc, 99:1 to 98:2) to give compound **14b** (59.4 mg, 45%) as a colourless oil. R_f_ **=** 0.77 (cyclohexane/EtOAc 8:2). ^1^H NMR (CDCl_3_, 300 MHz), δ (ppm): 3.84 (m, 1H, H_c_), 2.50 (ddd, *J* = 9.2 Hz, *J* = 5.7 Hz, *J* = 2.8 Hz, 1H, H_b_), 1.79-1.15 (m, 36H, H_d_-H_l_, H_a’_-H_i’_), 0.99 (t, 9H, *J* = 8.0 Hz, Si(CH_2_C*H*_3_)_3_), 0.88 (t, 6H, *J* = 6.8 Hz, H_m_, H_j’_), 0.67 (q, 6H, *J* = 8.0 Hz, Si(C*H*_2_CH_3_)_3_). ^13^C NMR (CDCl_3_, 75 MHz) δ (ppm): 177.0 (C_a_), 73.9 (C_c_), 50.8 (C_b_), 35.6, 32.1, 29.7, 29.6, 29.5, 29.4, 27.7, 25.1, 22.8 (18C, C_d_-C_l_, C_a’_-C_i’_), 14.3 (2C, C_m_, C_j’_), 6.9 (Si(CH_2_*C*H_3_)_3_), 5.1 (Si(*C*H_2_CH_3_)_3_). HRMS (ESI): calcd for C_29_H_60_NaO_3_Si [M+Na]^+^ 507.4204, found 507.4180. ${[\alpha]}_{D}^{20}$ **=** + 6 (*c* 1.02, CHCl_3_).

**(*R*)-3-Methoxy-octadecanoic acid 12**

Proton sponge® (272 mg, 1.27 mmol, 4 equiv) and Me_3_O**·**BF_4_ (188 mg, 1.27 mmol, 4 equiv) were added to a solution of **10b** (55) (100 mg, 0.32 mmol) in dry CH_2_Cl_2_ (1.5 mL). The reaction mixture was stirred for 24 h at room temperature then quenched by an aqueous solution of HCl 1N to acidify the solution to pH≈1. The solution was then filtered and washed by small portions of CH_2_Cl_2_. The filtrate was diluted with CH_2_Cl_2_ and washed twice by an aqueous solution of HCl 1N and by a saturated aqueous solution of NaCl. The organic layer was dried over Na_2_SO_4_, filtered and concentrated under reduced pressure. The residue was purified by flash silica gel chromatography (cyclohexane/EtOAc 98:2) to give the expected *O*-methylated derivative (86 mg, 83%).^1^H NMR (CDCl_3_, 300 MHz), δ (ppm): 3.68 (s, 3H, -OCH_3_), 3.62 (m, 1H, H_c_), 3.33 (s, 3H, C(=O)-OCH_3_), 2.53 (dd, 1H, *J =* 15.2 Hz, *J =* 7.3 Hz, H_b_), 2.40 (dd, 1H, *J =* 15.2 Hz, *J =* 5.4 Hz, H_b_’), 1.61-1.13 (m, 28H, H_d-_H_q_), 0.87 (t, 3H, *J =* 6.8 Hz, H_r_); ^13^C NMR (CDCl_3_, 75 MHz) ; δ (ppm): 172.6 (C_a_), 78.0 (C_c_), 57.2 (C(=O)-OCH_3_), 51.9 (-OCH_3_), 39.6 (C_b_), 39.6, 34.1, 32.2, 29.9, 29.6, 25.4, 23.0 (14C, C_d_-C_q_), 14.4 (C_r_); ESI HRMS: for C_20_H_40_NaO_3_ [M+Na]^+^: calcd 351.2870, found 351,2861. A 10% aqueous NaOH solution (3 mL) was added to a solution of this compound (95 mg, 0.29 mmol) in MeOH (3 mL). The reaction mixture was then stirred for 4 h at 40°C. Aqueous solution of HCl (6 M) was added to acidify the solution to pH ≈ 1. The solution was extracted three times with EtOAc and the combined organic layers were dried over Na_2_SO_4_, filtered and concentrated under reduced pressure. The desired product **12** (75 mg, 82%) was obtained as a colorless oil. ^1^H NMR (CDCl_3_, 300 MHz); δ (ppm): 3.67 (m, 1H, H_c_), 3.43 (s, 3H, -OCH_3_), 2.60 (dd, 1H, *J =* 15.6 Hz, *J =* 6.7 Hz, H_b_), 2.54 (dd, 1H, *J =* 15.6 Hz, *J =* 5.3 Hz, H_b_’), 1.74-1.16 (m, 28H, H_d_-H_q_), 0.92 (t, 3H, *J =* 6.8 Hz, H_r_); ^13^C NMR (CDCl_3_, 75 MHz); δ (ppm): 176.3 (C_a_), 77.8 (C_c_), 57.1 (C(=O)-OCH_3_), 39.2 (C_b_), 39.2, 33.7, 32.2, 29.9, 29.8, 29.6, 25.2, 22.9 (14C, C_d_-C_q_), 14.36 (C_r_); ESI HRMS: for C_19_H_38_NaO_3_ [M+Na]^+^: calcd 337.2713, found 337.2701.

**6-*O*-((2*R*, 3*R*)-2-Decyl-3-hydroxy-tridecanoyl)-α,α-d-trehalose 1**

A solution of compound **15**(62) (183 mg, 0.24 mmol, 2 equiv) in DCM (3 mL) was added to compound **14b** (57.3 mg, 0.12 mmol). EDCI (45.3 mg, 0.24 mmol, 2 equiv) and DMAP (28.9 mg, 0.24 mmol, 2 equiv) were then added and the reaction mixture was stirred at room temperature overnight. Saturated aqueous NaCl solution was added, and the reaction mixture was extracted three times with DCM. The combined organic layers were dried over Na_2_SO_4_ and concentrated under reduced pressure. The residue was purified by flash silica gel chromatography (cyclohexane/EtOAc, 99:1 to 90:10) to give the expected mono-*O*-esterified product (45.9 mg, 31%) as a colourless oil. R_f_ **=** 0.58 (cyclohexane/EtOAc 9:1). ^1^H NMR (CDCl_3_, 300 MHz) δ (ppm): 4.90 (d, 1H, *J =* 3.1 Hz, H_1_ or H_1’_), 4.84 (d, 1H, *J* = 3.1 Hz, H_1’_ or H_1_), 4.35 (dd, 1H, *J =* 11.7 Hz, *J =* 2.1 Hz, H_6a_), 4.05 (dd, 1H, *J* = 11.7 Hz, *J* = 3.7 Hz H_6b_), 4.01-3.80 (m, 5H, H_c_, H_3_, H_3’_, H_5_, H_5’_), 3.71-3.66 (m, 2H, H_6a’_, H_6b’_), 3.50 (at, 1H, *J* = 8.9 Hz, H_4_ or H_4’_), 3.47 (at, 1H, *J* = 8.8 Hz, H_4’_ or H_4_), 3.42 (dd, 1H, *J* = 9.3 Hz, *J* = 3.1 Hz, H_2_ or H_2’_), 3.38 (dd, 1H, *J* = 9.3 Hz, *J* = 3.1 Hz, H_2’_ or H_2_), 2.53 (ddd, *J* = 10.6 Hz, *J* = 5.5 Hz, *J* = 3.2 Hz, 1H, H_b_), 1.80-1.09 (m, 36H, H_d_-H_l_, H_a’_-H_i’_), 0.96 (t, 9H, *J* = 7.8 Hz, Si(CH_2_C*H*_3_)_3_), 0.88 (t, 3H, *J* = 6.6 Hz, H_m_ or H_j’_), 0.87 (t, 3H, *J* = 6.5 Hz, H_j’_ or H_m_), 0.60 (q, 6H, *J* = 7.9 Hz, Si(C*H*_2_CH_3_)_3_), 0.17-0.10 (m, 54H, 6 Si(CH_3_)_3_). ^13^C NMR (CDCl_3_, 75 MHz) δ (ppm): 174.2 (C_a_), 94.7, 94.6 (2C, C_1_, C_1’_), 73.6, 73.5, 73.4, 73.0, 72.9, 72.8 (6C, C_3_, C_3’_, C_5_, C_5’_, C_2_, C_2’_), 72.0, 71.5 (2C, C_4_, C_4’_), 70.8 (C_c_), 62.6 (C_6_), 61.8 (C_6’_), 52.5 (C_b_), 33.6 (C_d_), 32.1, 30.0, 29.9, 29.8, 29.7, 29.6, 29.5, 29.4, 28.3, 26.2, 25.4, 22.8 (17C, C_e_-C_l_, C_a’_-C_i’_), 14.3 (2C, C_m_, C_j’_), 7.1 (Si(CH_2_*C*H_3_)_3_), 5.2 (Si(*C*H_2_CH_3_)_3_), 1.2, 1.1, 1.0, 0.9, 0.3, 0.2 (6 Si(CH_3_)_3_). HRMS (ESI): calcd for C_59_H_128_NaO_13_Si_7_ [M+Na]^+^ 1263.7632, found 1263.7625. ${[\alpha]}_{D}^{20}$ **=** + 61 (*c* 0.83, CHCl_3_). To a solution of this compound (45.9 mg, 0.037 mmol) in MeOH (3.4 mL) were added 350 mg of Dowex 50WX8 (H^+^ form). The reaction mixture was stirred at room temperature for 1h15, then filtered, washed three times with MeOH and concentrated under reduced pressure. The residue was purified by flash silica gel chromatography (EtOAc/MeOH, 95:5 to 90:10) to give the desired TMM analog **1** (20 mg, 78%) as a colourless oil. R_f_ **=** 0.71 (EtOAc/MeOH, 10 mL, 7:3 + 2 drops of water). ^1^H NMR (CD_3_OD, 300 MHz) δ (ppm): 5.09 (d, 2H, *J =* 3.7 Hz, H_1_ and H_1’_), 4.62 (s, 1H, OH), 4.47 (dd, 1H, *J =* 11.8 Hz, *J =* 1.7 Hz, H_6a_), 4.18 (dd, 1H, *J* = 11.8 Hz, *J* = 5.3 Hz H_6b_), 4.07 (ddd, 1H, *J* = 9.9 Hz, *J* = 5.3 Hz, *J* = 1.7 Hz, H_5_) 3.87-3.75 (m, 4H, H_5’_, H_6a’_, H_3_, H_3’_), 3.73-3.64 (m, 2H, H_6b’_, H_c_), 3.48 (dd, 2H, *J* = 9.7 Hz, *J* = 3.7 Hz, H_2_ and H_2’_), 3.39-3.32 (m, 2H, H_4_, H_4’_), 2.43 (ddd, 1H, *J* = 10.2 Hz, *J* = 7.3 Hz, *J* = 4.1 Hz, H_b_), 1.70-1.17 (m, 36H, H_d_-H_l_, H_a’_-H_i’_), 0.90 (t, 6H, *J* = 6.9 Hz, H_m_ and H_j’_). ^13^C NMR (CD_3_OD, 75 MHz) δ (ppm): 176.2 (C_a_), 95.3, 95.2 (2C, C_1_, C_1’_), 74.4, 74.3, 73.8 (3C, C_3_, C_3’_, C_5’_), 73.6 (C_c_), 73.2 (2C, C_2_, C_2’_), 72.0, 71.9 (2C, C_4_, C_4’_), 71.4 (C_5_), 64.4 (C_6_), 62.6 (C_6’_), 54.2 (C_b_), 35.6, 33.1, 30.8, 30.7, 30.6, 30.5, 29.8, 29.7, 28.6, 26.6, 23.8 (18C, C_d_-C_l_, C_a’_-C_i’_), 14.5 (2C, C_m_, C_j’_). HRMS (ESI): calcd for C_35_H_66_NaO_13_ [M+Na]^+^ 717.4396, found 717.4365. ${[\alpha]}_{D}^{20}$ **=** + 93 (*c* 1, CHCl_3_).

**6-*O*-((2*R*, 3*R*)-2-Pentyl-3-hydroxy-octanoyl)-α,α-d-trehalose 2**

A solution of compound **14a** (46 mg, 0.13 mmol) in DCM (0.5 mL) was added to a solution of dried EDCI (32.6 mg, 0.26 mmol, 2 equiv) and DMAP (12.8 mg, 0.07 mmol, 0.5 equiv) in DCM (0.4 mL). A solution of compound **15** (62) (103.3 mg, 0.13 mmol, 1 equiv) in DCM (1.2 mL) was added and the reaction mixture was stirred at room temperature for 4 h. Then 2 more equiv of EDCI and 0.5 equiv of DMAP were added and the solution was stirred at RT for 2 h. Finally, DCC (110.2 mg, 4 equiv) and DMAP (12.8 mg, 0.5 equiv) were added, and the reaction mixture was stirred at room temperature overnight then filtered, washed three times with cold DCM and concentrated under reduced pressure. The residue was purified by flash silica gel chromatography (cyclohexane/EtOAc, 99:1 to 98:2) to give the mono-*O*-esterified product (81.7 mg, 56%) as a colourless oil. R_f_ **=** 0.55 (cyclohexane/EtOAc 9:1). ^1^H NMR (CDCl_3_, 360 MHz) δ (ppm): 4.90 (d, 1H, *J =* 3.2 Hz, H_1_ or H_1’_), 4.84 (d, 1H, *J* = 3.2 Hz, H_1’_ or H_1_), 4.35 (dd, 1H, *J =* 11.9 Hz, *J =* 2.2 Hz, H_6a_), 4.06 (dd, 1H, *J* = 11.9 Hz, *J* = 3.6 Hz H_6b_), 4.01-3.94 (m, 2H, H_c_, H_5_), 3.90 (at, 1H, *J* = 9.0 Hz, H_3_ or H_3’_), 3.89 (t, 1H, *J* = 9.0 Hz, H_3’_ or H_3_), 3.83 (td, 1H, *J* = 9.4 Hz, *J* = 3.5 Hz, H_5’_), 3.71-3.66 (m, 2H, H_6a’_, H_6b’_), 3.50 (at, 1H, *J* = 9.0 Hz, H_4_ or H_4’_), 3.46 (at, 1H, *J* = 9.0 Hz, H_4’_ or H_4_), 3.42 (dd, 1H, *J* = 9.0 Hz, *J* = 3.2 Hz, H_2_ or H_2’_), 3.38 (dd, 1H, *J* = 9.0 Hz, *J* = 3.0 Hz, H_2’_ or H_2_), 2.54 (ddd, *J* = 10.8 Hz, *J* = 5.4 Hz, *J* = 3.2 Hz, 1H, H_b_), 1.75 (dd, 1H, *J* = 7.4 Hz, *J* = 5.4 Hz, OH), 1.65-1.16 (m, 16H, H_d_-H_g_, H_a’_-H_d’_), 0.96 (t, 9H, *J* = 7.9 Hz, Si(CH_2_C*H*_3_)_3_), 0.88 (t, 3H, *J* = 7.0 Hz, H_h_ or H_e’_), 0.87 (t, 3H, *J* = 6.7 Hz, H_e’_ or H_h_), 0.60 (q, 6H, *J* = 7.9 Hz, Si(C*H*_2_CH_3_)_3_), 0.17-0.10 (m, 54H, 6 Si(CH_3_)_3_). ^13^C NMR (CDCl_3_, 90 MHz) δ (ppm): 174.1 (C_a_), 94.6, 94.5 (2C, C_1_, C_1’_), 73.6, 73.5, 73.4 (3C, C_3_, C_3’_, C_5_), 73.0, 72.9, 72.8 (3C, C_2_, C_2’_, C_5’_), 72.0, 71.5 (2C, C_4_, C_4’_), 70.8 (C_c_), 62.6 (C_6_), 61.8 (C_6’_), 52.5 (C_b_), 35.6, 32.1, 32.0, 28.0, 26.1, 25.0, 22.8, 22.7 (8C, C_d_-C_g_, C_a’_-C_d’_), 14.2 (2C, C_h_, C_e’_), 7.1 (Si(CH_2_*C*H_3_)_3_), 5.2 (Si(*C*H_2_CH_3_)_3_), 1.2, 1.1, 1.0, 0.3, 0.2 (6 Si(CH_3_)_3_). HRMS (ESI): calcd for C_49_H_108_NaO_13_Si_7_ [M+Na]^+^ 1123.6067, found 1123.6012. ${[\alpha]}_{D}^{20}$ **=** + 59 (*c* 0.9, CHCl_3_). To a solution of this compound (81 mg, 0.07 mmol) in MeOH (4 mL) were added 200 mg of Dowex 50WX8 (H^+^ form). The reaction mixture was stirred at room temperature for 1h15, then filtered, washed three times with MeOH and concentrated under reduced pressure. The residue was purified by flash silica gel chromatography (EtOAc/MeOH, 90:10) to give the desired product **2** (35 mg, 86%) as a colourless oil. R_f_ **=** 0.32 (EtOAc/MeOH, 10 mL, 7:3 + 2 drops of water). ^1^H NMR (CD_3_OD, 360 MHz) δ (ppm): 5.05 (d, 2H, *J =* 3.6 Hz, H_1_ and H_1’_), 4.44 (dd, 1H, *J =* 11.8 Hz, *J =* 1.9 Hz, H_6a_), 4.14 (dd, 1H, *J* = 11.8 Hz, *J* = 5.0 Hz, H_6b_), 4.03 (ddd, 1H, *J* = 10.1 Hz, *J* = 5.0 Hz, *J* = 1.9 Hz, H_5_) 3.83-3.74 (m, 4H, H_3_, H_3’_, H_5’_, H_6a’_), 3.68-3.62 (m, 2H, H_6b’_, H_c_), 3.45 (dd, 2H, *J* = 9.6 Hz, *J* = 3.5 Hz, H_2_, H_2’_), 3.36-3.26 (m, 2H, H_4_, H_4’_), 2.41 (ddd, 1H, *J* = 10.7 Hz, *J* = 6.8 Hz, *J* = 4.7 Hz, H_b_), 1.65-1.19 (m, 16H, H_d_-H_g_, H_a’_-H_d’_), 0.89 (t, 3H, *J* = 6.9 Hz, H_h_ or H_e’_), 0.87 (t, 3H, *J* = 6.9 Hz, H_e’_ or H_h_). ^13^C NMR (CD_3_OD, 90 MHz) δ (ppm): 176.1 (C_a_), 95.3, 95.2 (2C, C_1_, C_1’_), 74.4, 74.3, 73.8 (3C, C_3_, C_3’_, C_5_), 73.6 (C_c_), 73.2, 73.1 (2C, C_2_, C_2’_), 72.0, 71.9 (2C, C_4_, C_4’_), 71.4 (C_5’_), 64.3 (C_6_), 62.6 (C_6’_), 54.3 (C_b_), 35.6, 33.0, 32.9, 29.8, 28.3, 26.4, 23.7, 23.5 (8C, C_d_-C_g_, C_a’_-C_d’_), 14.5, 14.4 (2C, C_h_, C_e’_). HRMS (ESI): calcd for C_25_H_46_NaO_13_ [M+Na]^+^ 577.2831, found 577.2813. ${[\alpha]}_{D}^{20}$ **=** + 111 (*c* 0.86, MeOH/CHCl_3_ 3/1).

**6-*O*-(2-Tetradecyl-octadecanoyl)-α,α-d-trehalose 3**

To a solution of compound **15** (62) (242 mg, 0.31 mmol, 2 equiv) in DCM (1 mL) were added, 64 mg of DCC (0.45 mmol, 2 equiv) and 30 mg of DMAP (0.16 mmol, 1 equiv) and 75 mg of compound **8** (0.156 mmol, 1 equiv) diluted with 2 mL of DCM. The reaction mixture was stirred at room temperature for 6 h. Then, the mixture was diluted with DCM and a saturated aqueous NaCl solution was added. The resulted mixture was extracted three times with DCM. The combined organic layers were dried over Na_2_SO_4_ and concentrated under reduced pressure. The residue was filtered using flash silica gel chromatography (cyclohexane to cyclohexane: EtOAc, 13:1) to give the esterified intermediate (41.4 mg, 21%). ^1^H NMR (CDCl_3_, 300 MHz) δ (ppm): 4.92 (d, 1H, *J =* 3.2 Hz, H_1_ or H_1’_), 4.85 (d, 1H, *J* = 3.0 Hz, H_1’_ or H_1_), 4.50 (dd, 1H, *J =* 12.8 Hz, *J =* 3.1 Hz, H_6a_), 4.03-3.95 (m, 2H, H_5_, H_6b_), 3.90 (at, 1H, *J* = 9.0 Hz, H_3_), 3.89 (at, 1H, J = 9.0 Hz, H_3’_), 3.83 (m, 1H, H_5’_), 3.75-3.61 (m, 2H, H_6a’_, H_6b’_), 3.49 (at, 1H, *J* = 9.0 Hz, H_4_), 3.47 (at, 1H, *J* = 9.0 Hz, H_4’_), 3.42 (dd, 1H, *J* = 9.0 Hz, *J* = 3.2 Hz, H_2_), 3.37 (dd, 1H, *J* = 9.0 Hz, *J* = 3.0 Hz, H_2’_), 2.35 (m, 1H, H_b_), 1.90-0.95 (m, 56H, 28 CH_2_), 0.88 (2 t, 6H, *J* = 6.9 Hz, 2 CH_3_), 0.20-0.07 (m, 54H, 6 Si(CH_3_)_3_); ^13^C NMR (CDCl_3_, 75 MHz) δ (ppm): 176.5 (CO), 94.6 (C_1_), 94.5 (C_1’_), 73.7, 73.5, 73.1, 73.0, 72.8, 72.1, 71.5, 71.1 (C_2_, C_2’_, C_3_, C_3’_, C_4_, C_4’_, C_5_, C_5’_), 62.2 (C_6_), 61.8 (C_6’_), 46.0 (C_b_), 32.1, 29.9, 29.8, 29.7, 29.5, 29.4, 27.6, 27.5 (28C, C_c_-C_o_, C_a’_-C_o’_), 14.3 (2C, C_p_, C_p’_), 1.2, 1.1, 1.0, 0.3, 0.2 (6 Si(CH_3_)_3_). HRMS (ESI): calcd for C_62_H_132_NaO_12_ [M+Na]^+^ 1259.8227, found 1259.8184. The filtered derivative was therefore diluted with MeOH (5 mL), and 100 mg of Dowex 50WX8 (H^+^ form) was added to the solution. The reaction mixture was stirred at room temperature for 40 min, then filtered, washed three times with MeOH and concentrated under reduced pressure. The residue was purified by flash silica gel chromatography (DCM/MeOH, 85:15) to give the desired product **3** (21.1 mg, 77%) as a white solid. R_f_ **=** 0.26 (DCM/MeOH, 81:15). ^1^H NMR (CD_3_OD/CDCl_3_, 360 MHz) δ (ppm): 5.10 (m, 2H, H_1_, H_1’_), 4.39 (dd, 1H, *J* = 11.9 Hz, *J* = 2.2 Hz, H_6a_), 4.22 (dd, 1H, *J* = 11.9 Hz, *J* = 4.3 Hz, H_6b_), 3.99 (ddd, 1H, *J* = 10.0 Hz, *J* = 4.3 Hz, *J* = 2.2 Hz, H_5_), 3.84-3.73 (m, 4H, H_3_, H_3’_, H_5’_, H_6a’_), 3.64 (dd, 1H, *J* = 12.2 Hz, *J* = 5.7 Hz, H_6b’_), 3.49 (dd, 1H, *J* = 9.7 Hz, *J* = 3.2 Hz, H_2_ or H_2’_), 3.48 (dd, 1H *J* = 10.0 Hz, *J* = 3.6 Hz, H_2’_ or H_2_), 3.42-3.30 (m, 2H, H_4_, H_4’_), 2.35 (m, 1H, H_b_), 1.65-1.1 (m, 56H, H_c_-H_o_ and H_a’_-H_o’_), 0.89 (2t, 6H, *J* = 6.8 Hz, H_p_ and H_p’_). ^13^C NMR (CD_3_OD/CDCl_3_, 75 MHz) δ (ppm): 178.1 (C_a_), 94.7, 94.6 (2C, C_1_, C_1’_), 74.3, 74.0, 73.3, 72.7, 72.7, 71.6; 71.4, 71.0 (8C, C_2_, C_2’_, C_3_, C_3’_, C_4_, C_4’_, C_5_, C_5’_), 63.7 (C_6_), 62.5 (C_6’_), 46.6 (C_b_), 33.0, 32.7, 30.5, 30.4, 30.3, 30.2, 28.2, 28.1, 23.5 (28C, C_c_-C_o_ and C_a’_-C_o’_), 14.5 (2C, C_p_, C_p’_). HRMS (ESI): calcd for C_44_H_84_NaO_12_ [M+Na]^+^ 827.5963, found 827.5854.

**6-*O*-((*R*)-3-Hydroxy-octadecanoyl)-α,α-d-trehalose 4**

A solution of compound **15** (62) (132 mg, 0.17 mmol, 1.2 equiv) in CH_2_Cl_2_ (1.5 mL) was added *via* cannula to a solution of **11** (58 mg, 0.14 mmol), EDCI (43 mg, 0.28 mmol, 2.0 equiv), and DMAP (34 mg, 0.28 mmol, 2.0 equiv) in CH_2_Cl_2_ (0.5 mL). The reaction mixture was stirred overnight at room temperature and then diluted by a saturated aqueous solution of NaCl. The solution was extracted three times by CH_2_Cl_2_ and the combined organic layers were dried over Na_2_SO_4_, filtered and concentrated under reduced pressure. The residue was purified by flash silica gel chromatography (cyclohexane/EtOAc 98:2 to 96:4) to give the esterified compound (80 mg, 49%) as a colorless oil. ^1^H NMR (CDCl_3_, 300 MHz); δ (ppm): 4.94 (d, 1H, *J* = 3.0 Hz, H_1_), 4.91 (d, 1H, *J* = 3.0 Hz, H_1’_), 4.30 (dd, 1H, *J* = 11.5 Hz, *J* = 1.7 Hz, H_6a_), 4.17 (m, 1H, H_c_), 4.09 (dd, 1H, *J* = 11.5 Hz, *J* = 4.8 Hz, H_6b_), 4.02 (m, 1H, H_5_), 3.97-3.88 (m, 2H, H_3_ and H_3_’), 3.86 (m, 1H, H_5_’), 3.78-3.64 (m, 2H, H_6a_’ and H_6b_’), 3.54-3.41 (m, 4H, H_2_, H_2_’, H_4_, H_4_’), 2.55 (dd, 1H, *J* = 7.0 Hz, *J* = 15.4 Hz, 1H of H_b_), 2.46 (dd, 1H, *J* = 6.2 Hz, *J* = 15.4 Hz, 1H of H_b_), 1.75 (at, 1H, *J* = 6.6 Hz, OH), 1.49 (m, 2H, m, H_d_), 1.40-1.19 (m, 24H, H_e_-H_q_), 0.96 (t, 9H, *J* = 7.9 Hz, Si(CH_2_C*H*_3_)_3_), 0.90 (t, 3H, *J* = 6.5 Hz, H_r_), 0.61 (q, 6H, *J* = 7.9 Hz, Si(C*H*_2_CH_3_)_3_), 0.21-0.10 (54H, m, 6 Si(CH_3_)_3_); ^13^C NMR (CDCl_3_, 75 MHz); δ (ppm): 171.8 (Ca), 94.5, 94.3 (C1, C1’), 73.4, 73.4, 72.9, 72.8, 72.6, 72.0, 71.4, (C2, C2’, C3, C3’, C4, C4’, C5’), 70.7 (C5), 69.2 (Cc), 63.4 (C6), 61.7 (C6’), 42.7 (Cb), 37.7 (Cd), 31.9, 29.7, 29.7, 29.6, 29.6, 29.4, 25.2, 22.7 (Ce-Cq), 14.1 (Cr), 7.0 (Si(CH_2_*C*H_3_)_3_), 4.9 (Si(*C*H_2_CH_3_)_3_), 1.0, 1.0, 0.9, 0.9, 0.2, 0.1 (Si(CH_3_)_3_); ESI HRMS: calcd for C_54_H_118_NaO_13_Si_7_[M+Na]^+^: 1193.6850, found 1193.6806. Dowex 50WX8 (H^+^ form) resin (700 mg) was added to solution of the esterified compound (78 mg, 0.07 mmol) in MeOH (5 mL). The solution was stirred for 40 min at room temperature, then filtered, washed with MeOH and concentrated under reduced pressure. The residue was purified by flash silica gel chromatography (CH_2_Cl_2_/MeOH 95:5 to 90:10) to give compound **4** (41 mg, quant.) as a white solid. ^1^H NMR (CD_3_OD /CDCl_3_, 360 MHz) δ (ppm): 5.11, 5.10 (2d, 2H, *J =* 3.8 Hz, H_1_ and H_1’_), 4.45 (dd, 1H, *J =* 11.9 Hz, *J =* 2.0 Hz, H_6a_), 4.22 (dd, 1H, *J =* 11.9 Hz, *J =* 5.4 Hz, H_6b_), 4.09 (m, 1H, H_5_), 4.02 (m, 1H, H_c_), 3.84-3.78 (m, 4H, H_3_, H_3’_, H_6a’_, H_5’_), 3.70 (dd, 1H, *J* = 12.0 Hz, *J* = 5.7 Hz H_6b’_), 3.51 (dd, 1H, *J* = 9.7 Hz, *J* = 4.1 Hz, H_2_ or H_2’_), 3.50 (dd, 1H, *J* = 9.7 Hz, *J* = 3.8 Hz, H_2’_ or H_2_), 3.39-3.35 (m, 2H, H_4_, H_4’_), 2.55 (dd, 1H, *J =* 15.0 Hz, *J =* 4.6 Hz, 1H of H_b_), 2.45 (dd, 1H, *J =* 15.0 Hz, *J =* 8.4 Hz, 1H of H_b_), 1.54-1.24 (m, 28H, H_d_-H_q_), 0.93 (t, 3H, *J* = 6.8 Hz, H_r_). ^13^C NMR (CD_3_OD, 90 MHz) δ (ppm): 172.0 (C_a_), 93.9, 93.8 (2C, C_1_, C_1’_), 73.1 (C_5_), 72.5, 71.8 (2C, C_3_, C_3’_), 70.5 (2C, C_2_, C_2_’), 70.0 (2C, C_4_, C_4_’), 69.9 (C_5_’), 68.0 (C_c_), 63.2 (C_6_), 61.2 (C_6’_), 41.9 (C_b_), 36.7 (C_d_), 31.7, 29.4, 29.3, 29.1, 25.3, 22.3 (13C, C_e_-C_q_), 13.0 (C_r_). HRMS (ESI): calcd for C_30_H_56_NaO_13_ [M+Na]^+^ 647.3613, found 647.3614. ${[\alpha]}_{D}^{20}$ **=** +97 (*c* 1.27, CHCl_3_/MeOH 1/1).

**6-*O*-(*R*)-3-methoxy-octadecanoyl)- α,α-d-trehalose 5**

A solution of **15** (62) (171 mg, 0.22 mmol, 1.2 equiv) in CH_2_Cl_2_ (1.2 mL) was added *via* cannula to a solution of **12** (57 mg, 0.18 mmol), EDCI (56 mg, 0.36 mmol, 2 equiv), and DMAP (44 mg, 0.36 mmol, 2 equiv) in CH_2_Cl_2_ (0.5 mL). The reaction mixture was stirred overnight at room temperature and then diluted by a saturated aqueous solution of NaCl. The solution was extracted three times by CH_2_Cl_2_ and the combined organic layers were dried over Na_2_SO_4_, filtered and concentrated under reduced pressure. The residue was purified by flash silica gel chromatography (cyclohexane/EtOAc 98:2 to 92:8) to give the desired esterified compound (67 mg, 35%) as a colorless oil. ^1^H NMR (CDCl_3_, 300 MHz); δ (ppm): 4.93 (d, 1H, *J =* 3.1 Hz, H_1’_), 4.91 (d, 1H, *J* = 3.1 Hz, H_1_), 4.34 (dd, 1H, *J =* 2.0 Hz, *J =* 11.7 Hz, H_6a_), 4.10 (dd, 1H, *J =* 5.0 Hz, *J =* 11.7 Hz, H_6b_), 4.02 (ddd, 1H, *J =* 2.0 Hz, *J =* 5.0 Hz, *J =* 9.0 Hz, H_5_), 3.93 (at, 1H, *J =* 9.0 Hz, H_3_), 3.91 (at, 1H, *J =* 9.0 Hz, H_3’_), 3.85 (m, 1H, H_5’_), 3.77-3.61 (m, 3H, H_6’a_, H_6’b_ and H_c_), 3.49 (at, 1H, *J =* 9.0 Hz, H_4_), 3.46 (at, 1H, *J =* 9.0 Hz, H_4’_), 3.45 (dd, 1H, *J =* 3.1 Hz, *J =* 9.0Hz, H_2_), 3.44 (dd, 1H, *J =* 3.1 Hz, *J =* 9.0 Hz, H_2’_), 3.36 (s, 3H, -OCH_3_), 2.63 (dd, 1H, *J =* 6.9 Hz, *J =* 15.8 Hz, 1H of H_b_), 2.44 (dd, 1H, *J =* 5.6 Hz, *J =* 15.8 Hz, 1H of H_b_) 1.58-1.20 (m, 28H, H_d_-H_q_), 0.18-0.13 (6s, 54H, 6 Si(CH_3_)_3_) ; ^13^C NMR (CDCl_3_, 75 MHz); δ (ppm): 172.0 (C_a_), 94.7 (C_1_ or C_1_’), 94.5 (C_1_ or C_1_’), 77.4 (C_c_), 73.7, 73.5, 73.1, 73.0, 72.8, 72.2, 71.6, 70.1 (8C, C_2_, C_2_’, C_3_, C_3_’, C_4_, C_4_’, C_5_, C_5_’), 63.7 (C_6_), 61.9 (C_6_’), 57.2 (O-CH_3_), 39.4 (C_b_), 34.2, 32.1, 29.9, 29.6, 25.3, 22.8 (14C, C_d_-C_q_), 14.3 (C_r_), 1.3, 1.2, 1.1, 1.1, 0.7, 0.3 (6 Si(CH_3_)_3_) ; ESI HRMS: calcd for C_49_H_106_NaO_13_Si_6_ [M+Na]^+^: 1093.6141, found 1093.6101. Dowex 50WX8 (H^+^ form) resin (480 mg) was added to a solution of the esterified compound (51 mg, 0.05 mmol) in MeOH (4 mL). The solution was stirred for 1h at room temperature, then filtered, washed with MeOH and concentrated under reduced pressure. The residue was purified by flash silica gel chromatography (CH_2_Cl_2_/MeOH 95:5 to 90:10) to give the desired product **5** (24 mg, quant.) as a white solid. ^1^H NMR (CD_3_OD, 250 MHz); δ (ppm): 5.12 (d, 1H, *J =* 3.6 Hz, H_1_), 5.10 (d, 1H, *J =* 3.6 Hz, H_1’_), 4.41 (dd, 1H, *J =* 2.7 Hz, *J =* 11.9 Hz, H_6a_), 4.23 (dd, 1H, *J =* 5.1 Hz, *J =* 11.9 Hz, H_6b_), 4.07 (m, 1H, H_5_), 3.90-3.75 (m, 4H, H_3_, H_3’_, H_5’_ and H_6’a_), 3.75-3.62 (m, 2H, H_6’b_, H_c_), 3.55-3.45 (m, 2H, H_2_, H_2’_), 3.37 (s, 3H, O-CH_3_), 3.42-3.29 (m, 2H, H_4_, H_4’_), 2.58-2.50 (m, 2H, H_b_), 1.66-1.19 (m, 28H, H_d_-H_q_), 0.93 (t, 3H, *J =* 6.3 Hz, H_r_) ; ^13^C NMR (CD_3_OD, 62.5 MHz); δ (ppm): 171.9 (C_a_), 93.9, 93.8 (C_1_, C_1_’), 77.8 (C_c_), 73.2, 73.1, 72.5, 71.8, 70.5 (7C, C_2_, C_2_’, C_3_, C_3_’, C_4_, C_4_’, C_5_’), 70.0 (C_5_), 63.2 (C_6_), 61.3 (C_6_’), 55.9 (CH_3_-O), 38.8 (C_b_), 33.5, 31.7, 29.4, 29.3, 29.1, 24.8, 22.3 (14C, C_d_-C_q_), 13.1 (C_r_); ESI HRMS: calcd for C_31_H_58_NaO_13_ [M+Na]^+^: 661.3770, found 661.3751.

**6-*O*-(hexadecanoyl)-α,α-d-trehalose 6**

A solution of **15** (62) (248 mg, 0.32 mmol, 1.2 equiv) in CH_2_Cl_2_ (2 mL) was added *via* cannula to a solution of commercially available palmitic acid (70 mg, 0.27 mmol), EDCI (84 mg, 0.54 mmol, 2 equiv), and DMAP (66 mg, 0.54 mmol, 2 equiv) in CH_2_Cl_2_ (2 mL). The reaction mixture was stirred overnight at room temperature and then diluted by a saturated aqueous solution of NaCl. The solution was extracted three times by CH_2_Cl_2_ and the combined organic layers were dried over Na_2_SO_4_, filtered and concentrated under reduced pressure. The residue was purified by flash silica gel chromatography (cyclohexane/EtOAc 98:2 to 9:1) to give the esterified product (139 mg, 51%) as a colorless oil. ^1^H NMR (CDCl_3_, 400 MHz); δ (ppm): 4.94 (d, 1H, *J* = 2.9 Hz, H_1_), 4.93 (d, 1H, *J* = 2.9 Hz, H_1’_), 4.32 (dd, 1H, *J* = 11.9 Hz, *J* = 2.2 Hz, H_6a_), 4.08 (dd, 1H, *J* = 11.9 Hz, *J* = 4.3 Hz, H_6b_), 4.05-4.00 (m, 1H, H_5_), 3.93 (at, 1H, *J* = 9.0 Hz, H_3_), 3.91 (at, 1H, *J* = 9.0 Hz, H_3’_), 3.86 (m, 1H, H_5’_), 3.77-3.63 (m, 2H, H_6’a_ and H_6’b_), 3.50 (at, 1H, *J* = 9.0 Hz, H_4_), 3.49 (at, 1H, *J* = 9.0 Hz, H_4’_), 3.46 (dd, 1H, *J* = 9.0 Hz, 2.9 Hz, H_2_), 3.44 (dd, 1H, *J =* 9.0 Hz, *J =* 2.9 Hz, H_2’_), 2.40-2.33 (m, 2H, H_b_), 1.79 (m, 1H, OH), 1.70-1.19 (m, 26 H, H_c_-H_o_), 0.90 (t, 3H, *J =* 6.7 Hz, H_p_), 0.19-0.13 (6s, 54H, 6 Si(CH_3_)_3_); ^13^C NMR (CDCl_3_, 75 MHz); δ (ppm): 173.8 (C_a_), 94.5, 94.4 (C1, C1’), 73.5, 73.3, 73.0, 72.8, 72.6, 71.9, 71.4 (C2, C2’, C3, C3’, C4, C4’), 70.8 (C5), 63.3 (C6), 61.6 (C6’), 34.2 (Cb), 32.0, 29.7, 29.7, 29.6, 29.5, 29.4, 29.3, 29.2, 24.8, 22.7 (Cc-Co), 14.1 (Cp), 1.1, 1.0, 0.9, 0.2, 0.1 (6 Si(CH_3_)_3_); ESI HRMS: calcd for C_46_H_100_NaO_12_Si_6_ [M+Na]^+^: 1035.5723, found 1035.5718. Dowex 50WX8 (H^+^ form) resin (457 mg) was added to a solution of the esterified compound (139 mg, 0.14 mmol) in MeOH (6 mL). The solution was stirred for 40 min at room temperature, then filtered, washed with MeOH and concentrated under reduced pressure. The residue was purified by flash silica gel chromatography (CH_2_Cl_2_/MeOH 90:10 to 80:20) to give the desired product **6** (78 mg, 96%) as a white solid. NMR data were in agreement with literature (63). ^1^H NMR (CDCl_3/_CD_3_OD, 360 MHz); δ (ppm): 5.10 (d, 1H, *J* = 3.7 Hz, H_1_), 5.07 (d, 1H, *J* = 3.7 Hz, H_1’_), 4.36 (dd, 1H, *J* = 2.1 Hz, *J* = 11.9 Hz, H_6a_), 4.20 (dd, 1H, *J* = 5.1 Hz, *J* = 11.9 Hz, H_6b_), 4.02 (ddd, 1H, *J* = 2.1 Hz, *J* = 5.1 Hz, *J* = 10.0 Hz, H_5_), 3.85-3.74 (m, 4H, H_3_, H_3’_, H_6’a_ and H_6’b_), 3.67 ( m, 1H, H_5’_), 3.49 (dd, 1H, *J* = 3.6 Hz, *J* = 9.7 Hz, H_2_ or H_2’_), 3.46 (dd, 1H, *J* = 3.6 Hz, *J* = 9.8 Hz, H_2_ or H_2’_), 3.33 (m, 2H, H_4_ and H_4’_), 2.34 (t, 2H, *J* = 7.4 Hz, H_b_), 1.61 (m, 2H, H_c_), 1.38-1.23 (m, 24H, H_d_-H_o_), 0.9 (t, 3H, *J* = 6.9 Hz, H_p_); ^13^C NMR (CDCl_3_/CD_3_OD, 90 MHz); δ (ppm): 175.3 (Ca), 94.5, 94.4 (C1, C1’), 73.9, 73.7, 73.1 (C3, C3’, C5’), 72.5, 72.4 (C2, C2’), 71.3, 71.1 (C4, C4’), 70.7 (C5), 63.8 (C6), 62.2 (C6’), 34.7 (Cb), 32.5, 30.3, 30.2, 30.1, 30.0, 29.9, 29.8, 25.5, 23.3 (Cc-Co), 14.4 (Cp); ESI HRMS: calcd for C_28_H_52_NaO_12_[M+Na]^+^: 603.3351, found 603.3368.


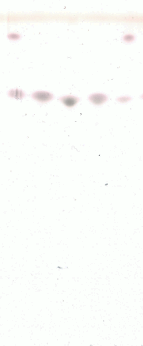

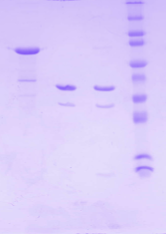


- TMM

- TDM

TDM/TMM mix

p TMM

s TMM

s TMM + pTMM

s TMM + pTDM

Front of migration

Start line

MytA

MytC

MytC

S189V

MW

**A**

**B**

- 70

- 50

- 35

- 15

- 25

**Fig. S1.** **Analysis of Myts enzymes and mycolate donors purified from *C. glutamicum*.** *A*, SDS/PAGE 12 % analysis of purified MytA_his_, MytC_his_, MytC _his_ (S189V) and_,_ visualized by Coomassie blue coloration. *B*, TLC of *C. glutamicum* lipid extract (TDM/TMM mix), purified TMM or TDM (pTMM, pTDM) and synthetic TMM (sTMM) developed in CHCl_3_-CH_3_OH-H_2_O (34:15:2 vol/vol/vol) revealed by sulfuric acid staining.


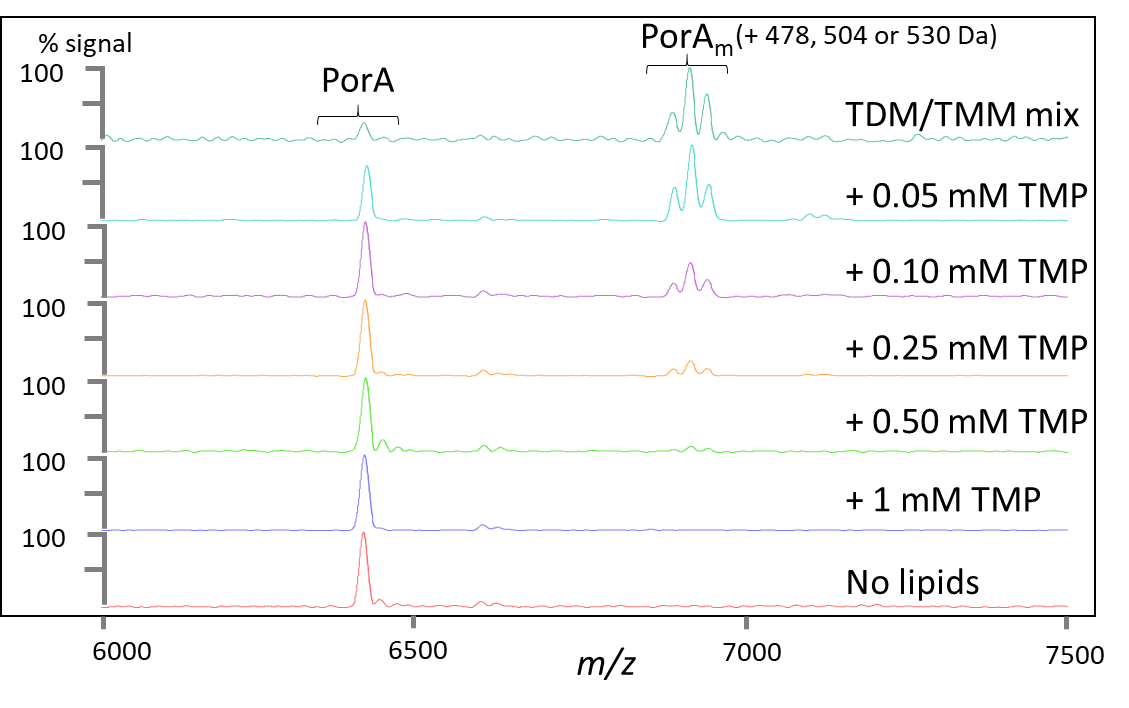


**Fig. S2.** **MytC inhibition by TMP (analog 6).** MALDI-TOF MS spectra of the PorA substrate (*m/z* 6410) leading to the formation of the expected major mycoloylated products PorAm (*m/z* 6888 (C32:0), 6914 (C34:1), 6940 (C36:2)) in the presence of MytC and increasing TMP concentration (0.05 to 1 mM)


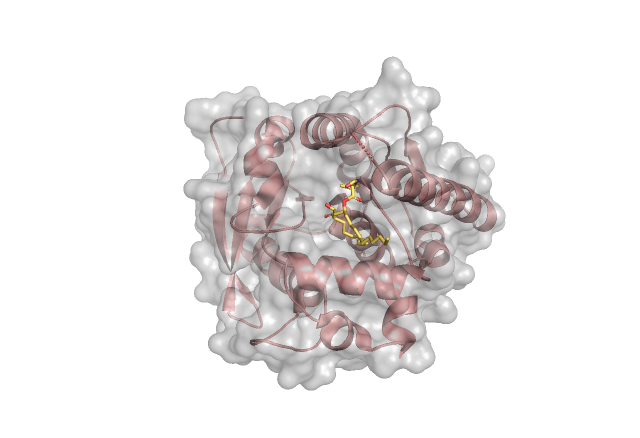

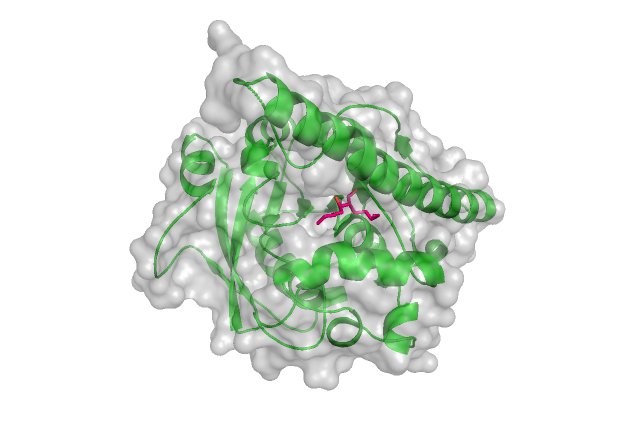

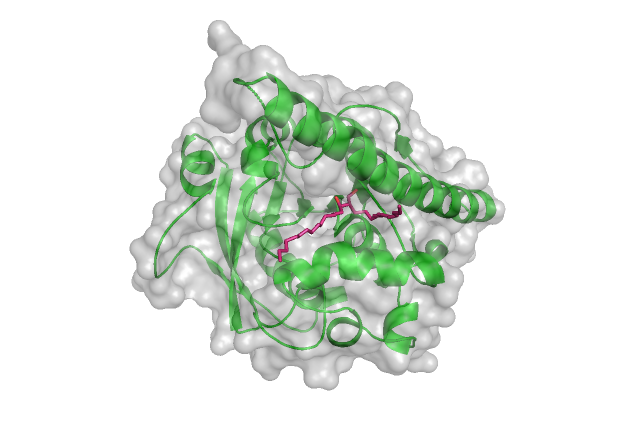


A

B

C


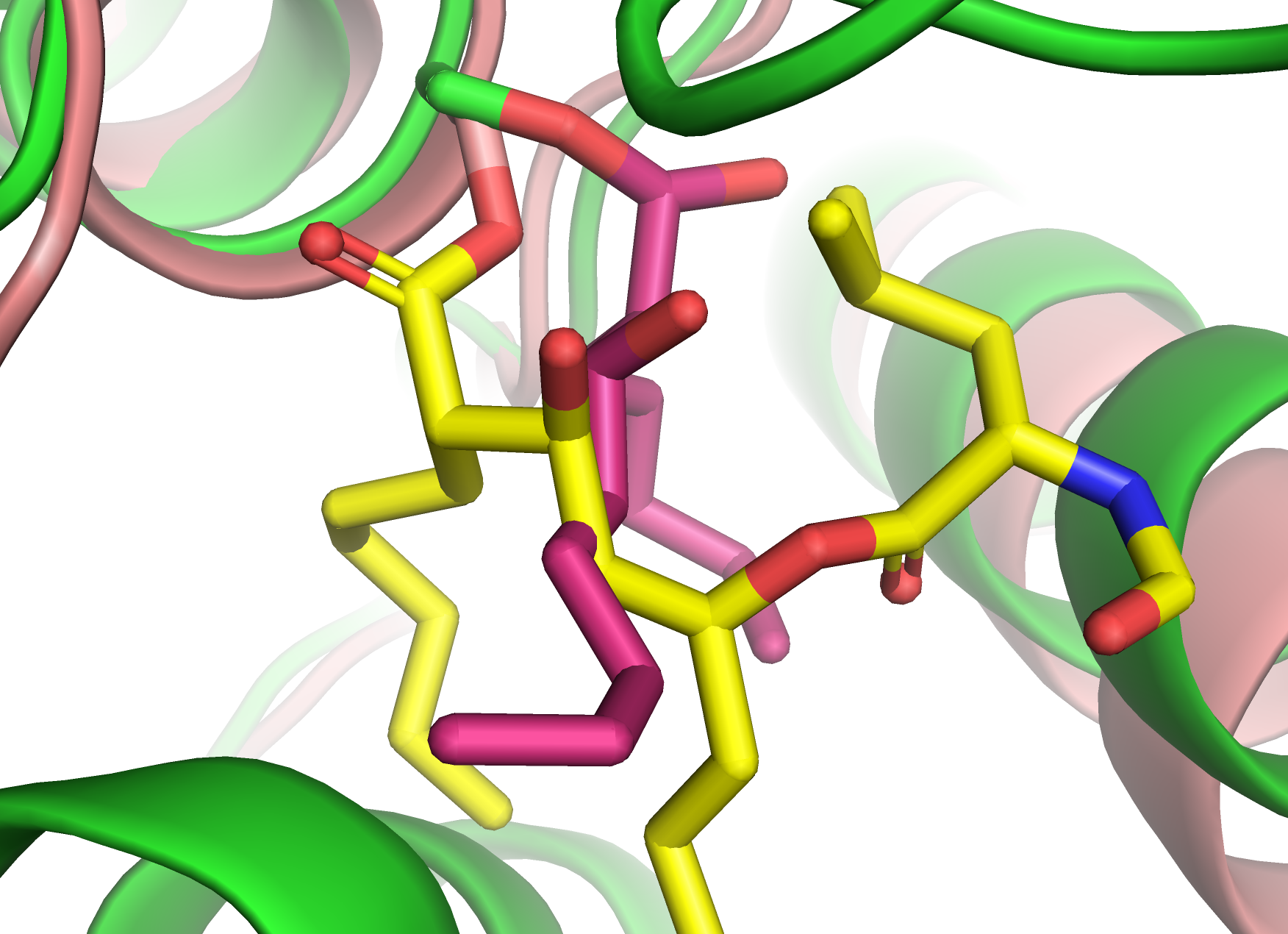


**Ag85C**

**Ser124 Oγ**

**C13:0 mycolate**

**THL**

**MytC**

**Ser189 Oγ**

**β-OH**

D

**Fig. S3. Alkyl pockets of mycoloyltransferases** (ribbon presentation imbedded in molecular surface). *A*, MytC-acyl-enzyme: crystal structure of MytC covalently bound to TMM-C13:0 (red sticks). *B*, Model of a MytC-acyl-enzyme bound to a natural mycoylate (red sticks). The model was obtained by replacing the short alkyl chains of TMM-C13:0 by long chains. *C*, Ag85C-acyl-enzyme obtained with THL (sticks) (pdbcode 1VA5) Ag85C is shown in the same orientation as MytC in panels *A* and *B*. *D*, detailed view of the superposition of the acylated serines from the MytC and Ag85C acyl-enzymes. The β-OH position of both molecules (THL and TMM-C13:0) is also indicated.


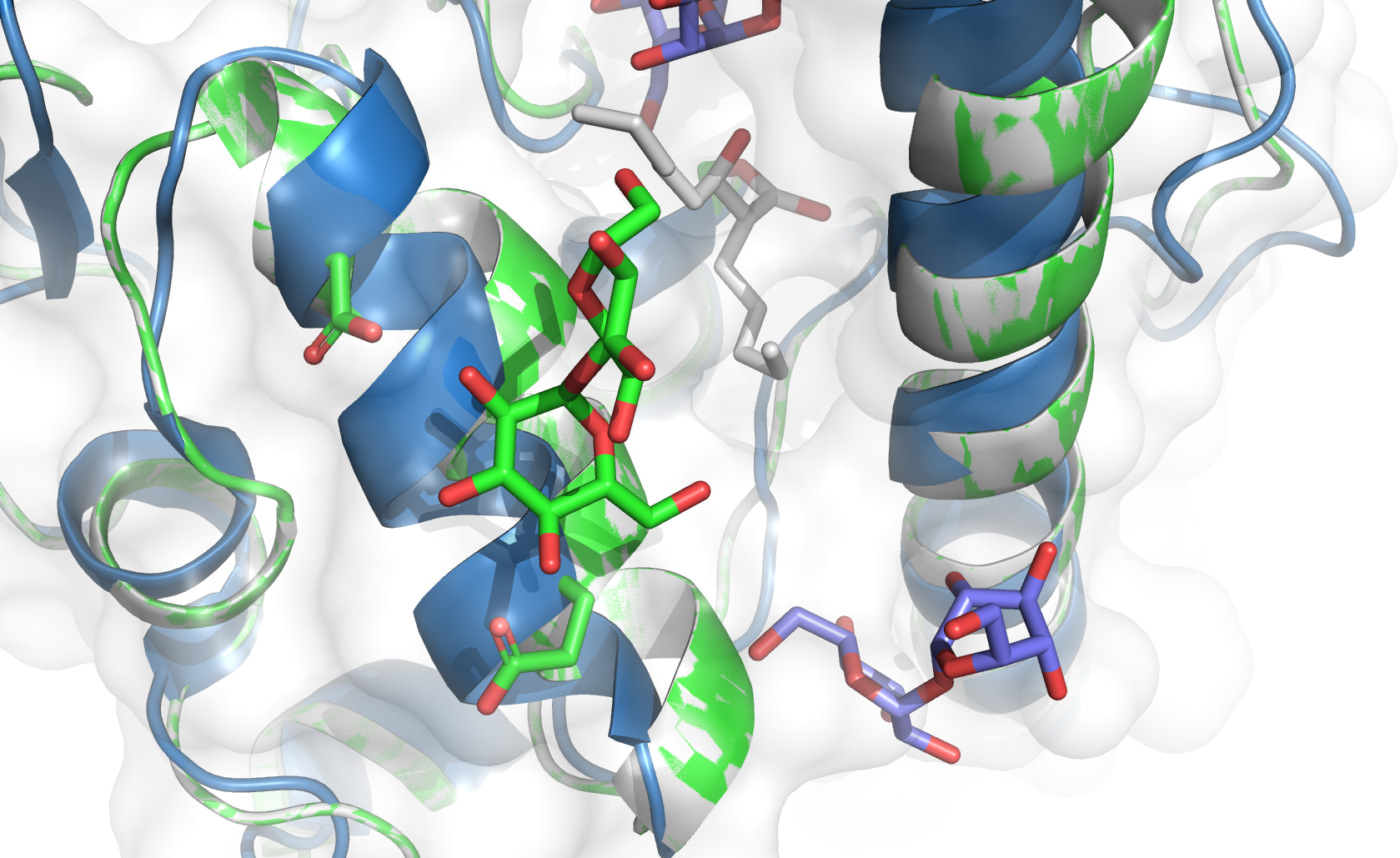


**D232**

**E225**

3.0Å

2.9Å

**𝛼8**

**𝛼11**

Trehalose surface pocket

**A**


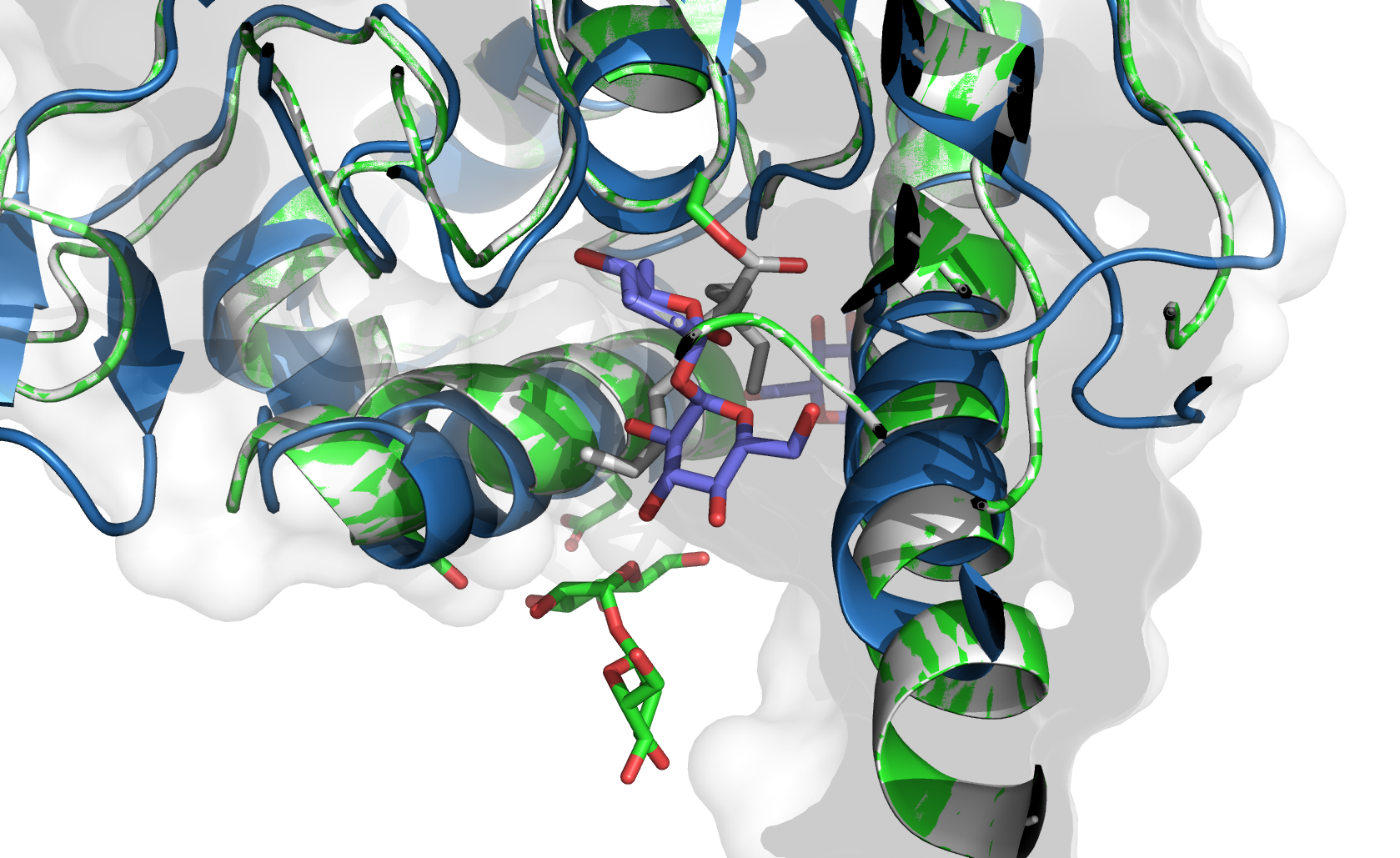


**𝛼8**

**𝛼11**

Trehalose surface pocket

**D232**

**E225**

TMM-C13:0

Trehalose

**B**

**Fig. S4. Two perpendicular views of superposed trehalose binding sites in MytC (green) and Ag85 (palecyan).** Trehalose bound to MytC is in green sticks, the acyl-intermediate in MytC is in grey sticks, trehaloses bound to Ag85 are in violet sticks. Polar interactions of MytC with trehalose are shown as dotted lines (on the left pannel).

**
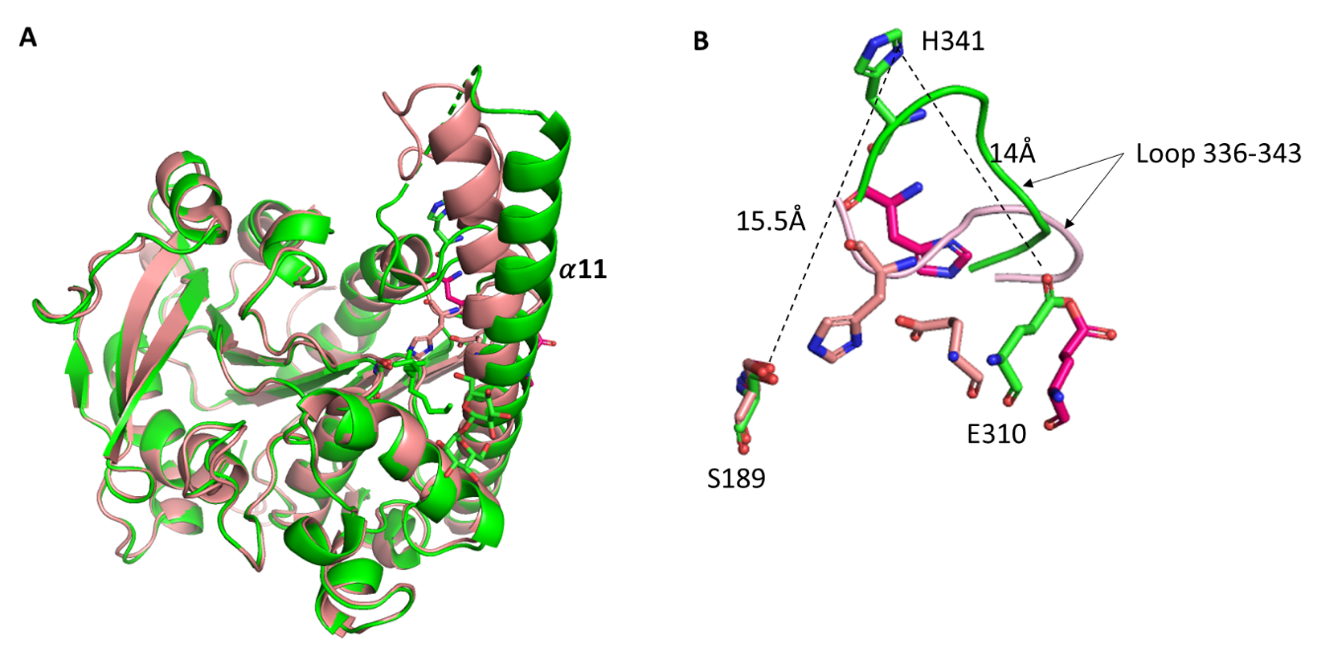
**

**Fig. S5. MytC structural changes between the apo and acyl-enzyme intermediate forms**. *A*, Superposition of MytC model AlphaFold in wheat and MytC-acyl-enzyme in green. *B*, MytC catalytic triad residues (S189, H341 and E310): MytC model AlphaFold in wheat, MytC apo enzyme in magenta, MytC-acyl-enzyme in green showing the histidine displacement.


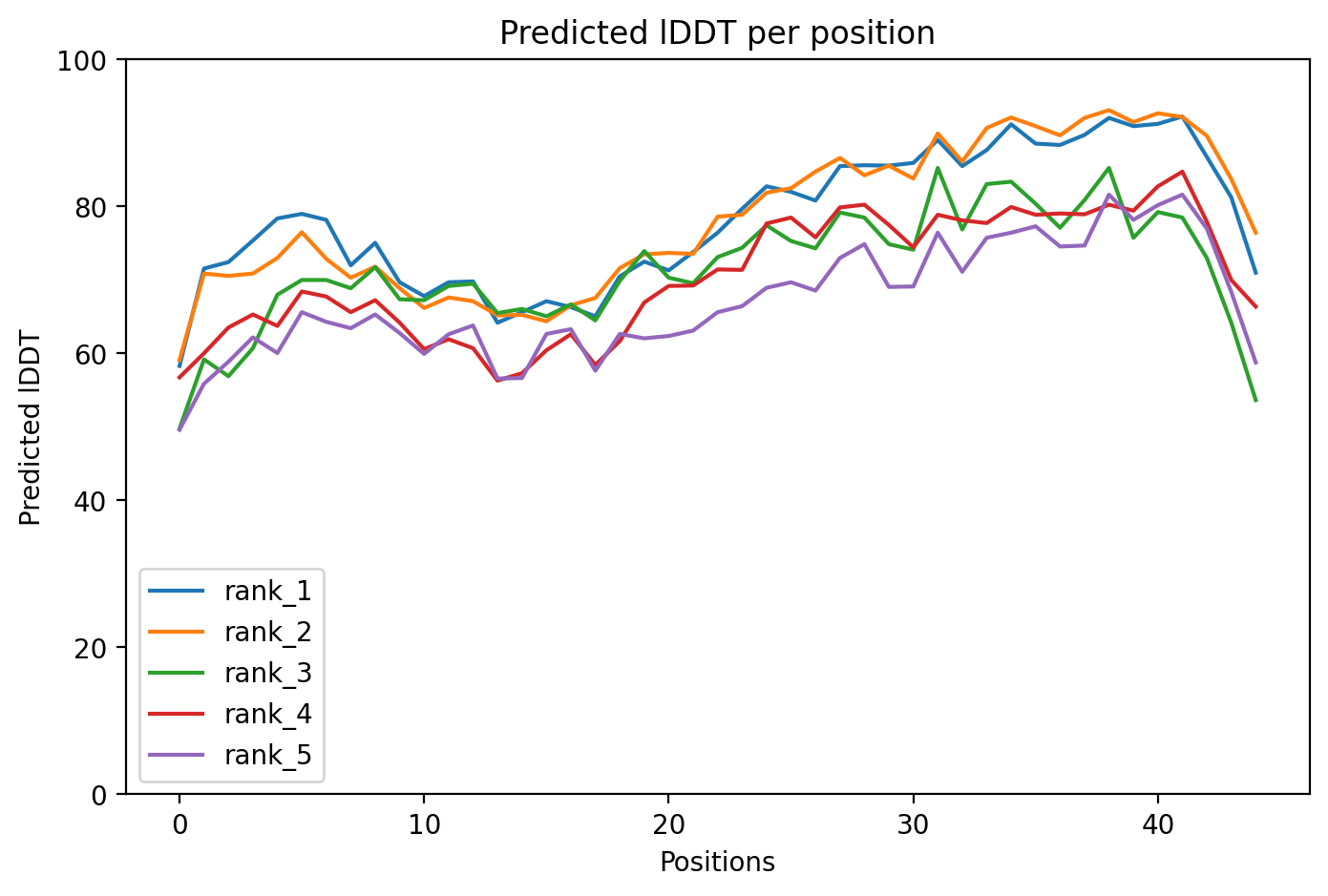

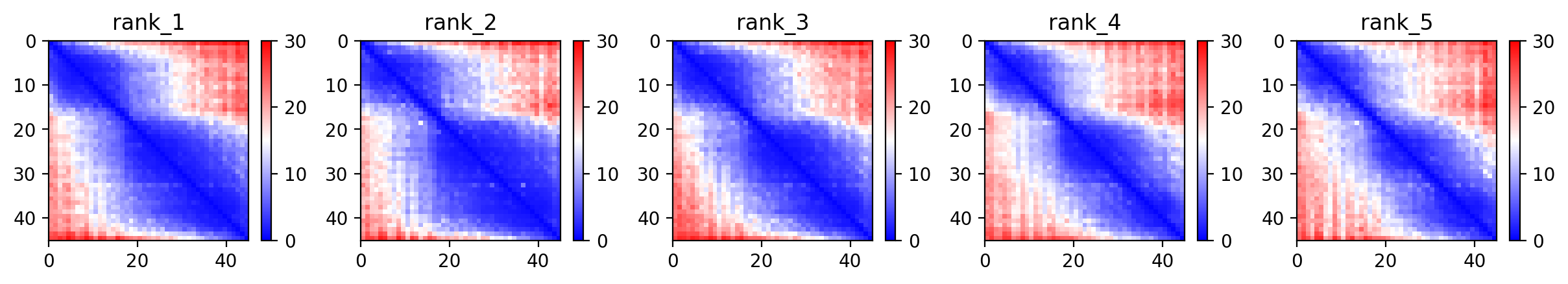

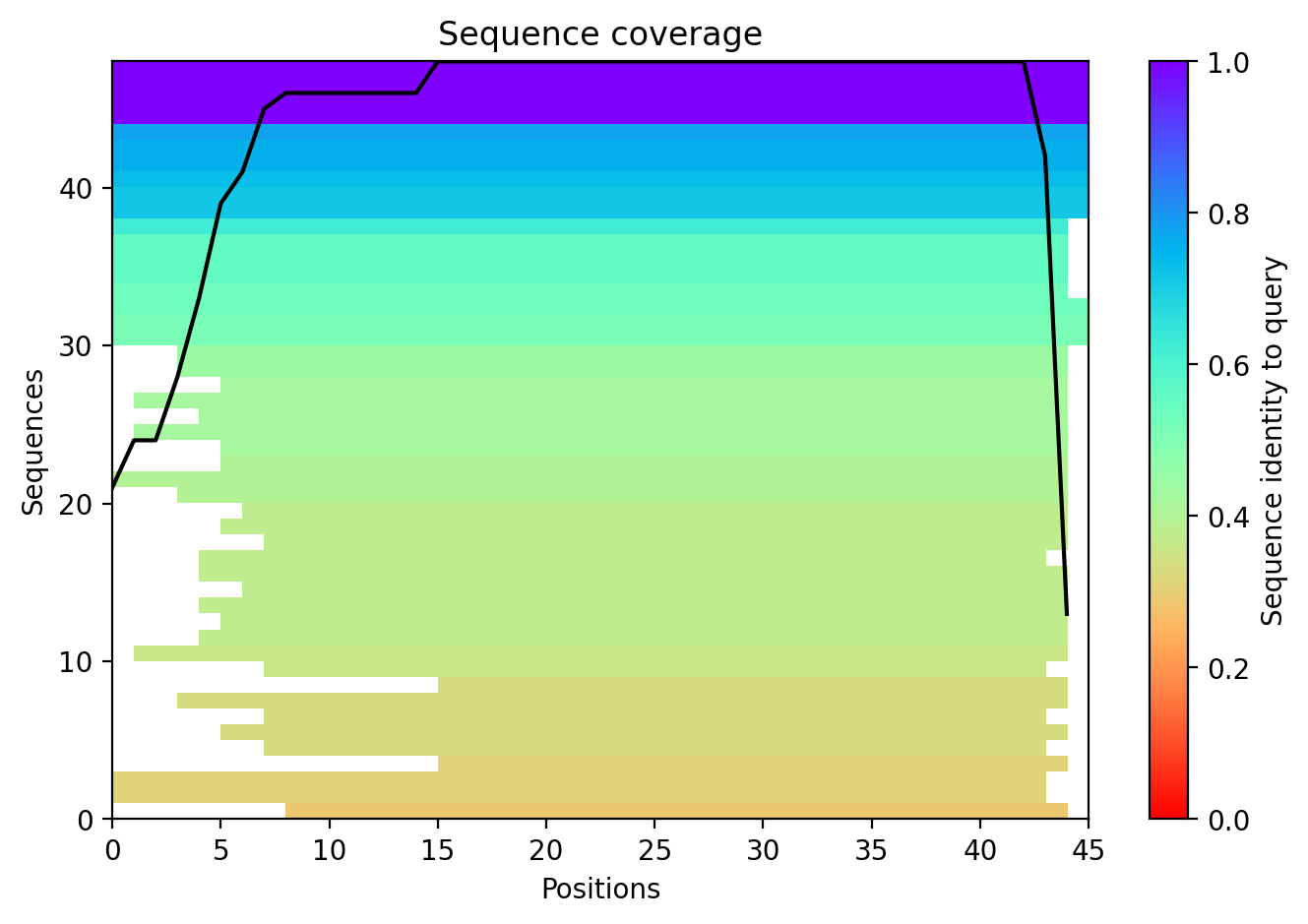

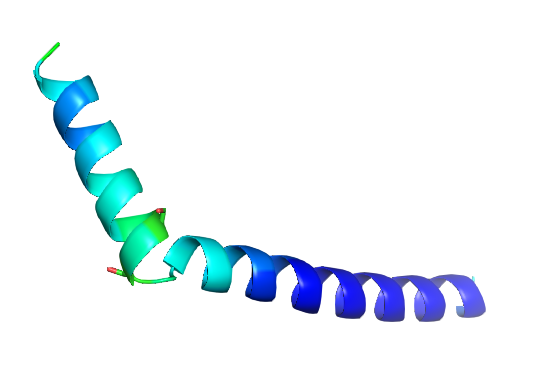


S15

S17

Predicted alignement error

A

Predicted IDDT per position

Sequence coverage

B

C

D

**Fig. S6. PorA model prediction.** (A) PorA model (color by model confidence). (B,C,D) Statistic of AlphaFold model prediction for PorA.


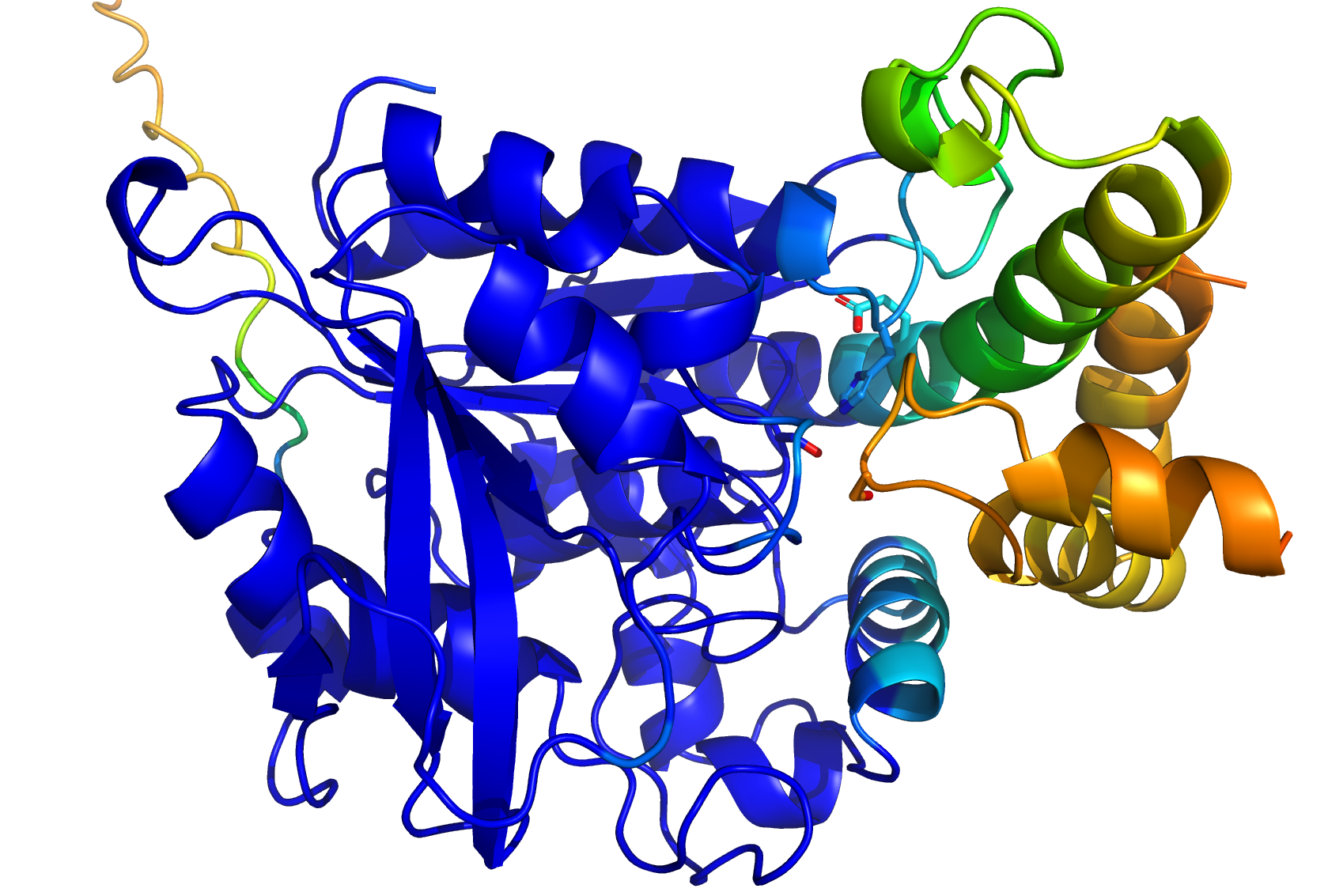


**S15**

**S189**

**H341**

**E310**

**C**

N

C

N


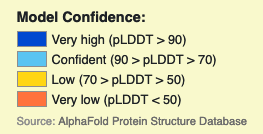


**PorA**

**MytC**

Predicted alignement error


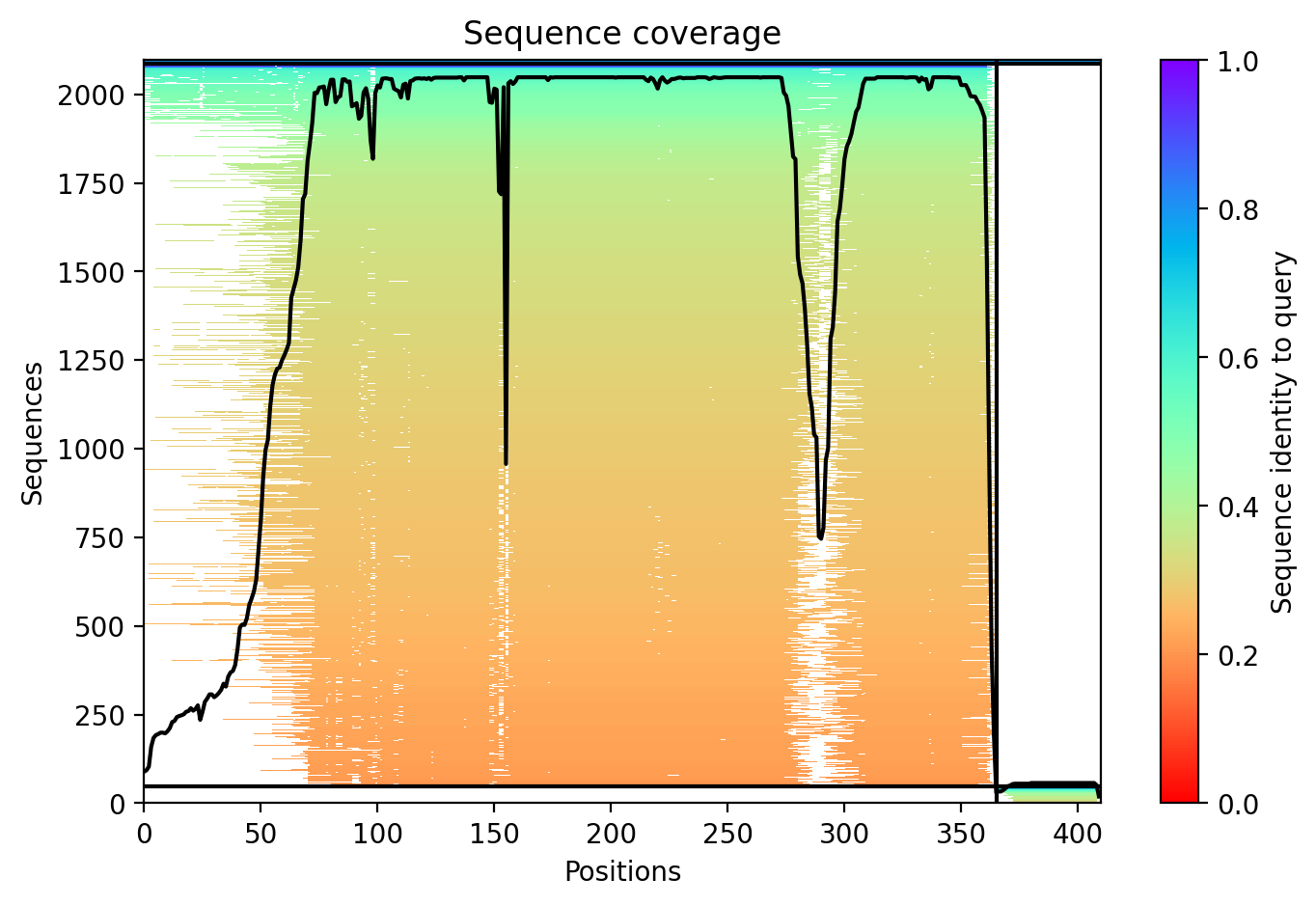

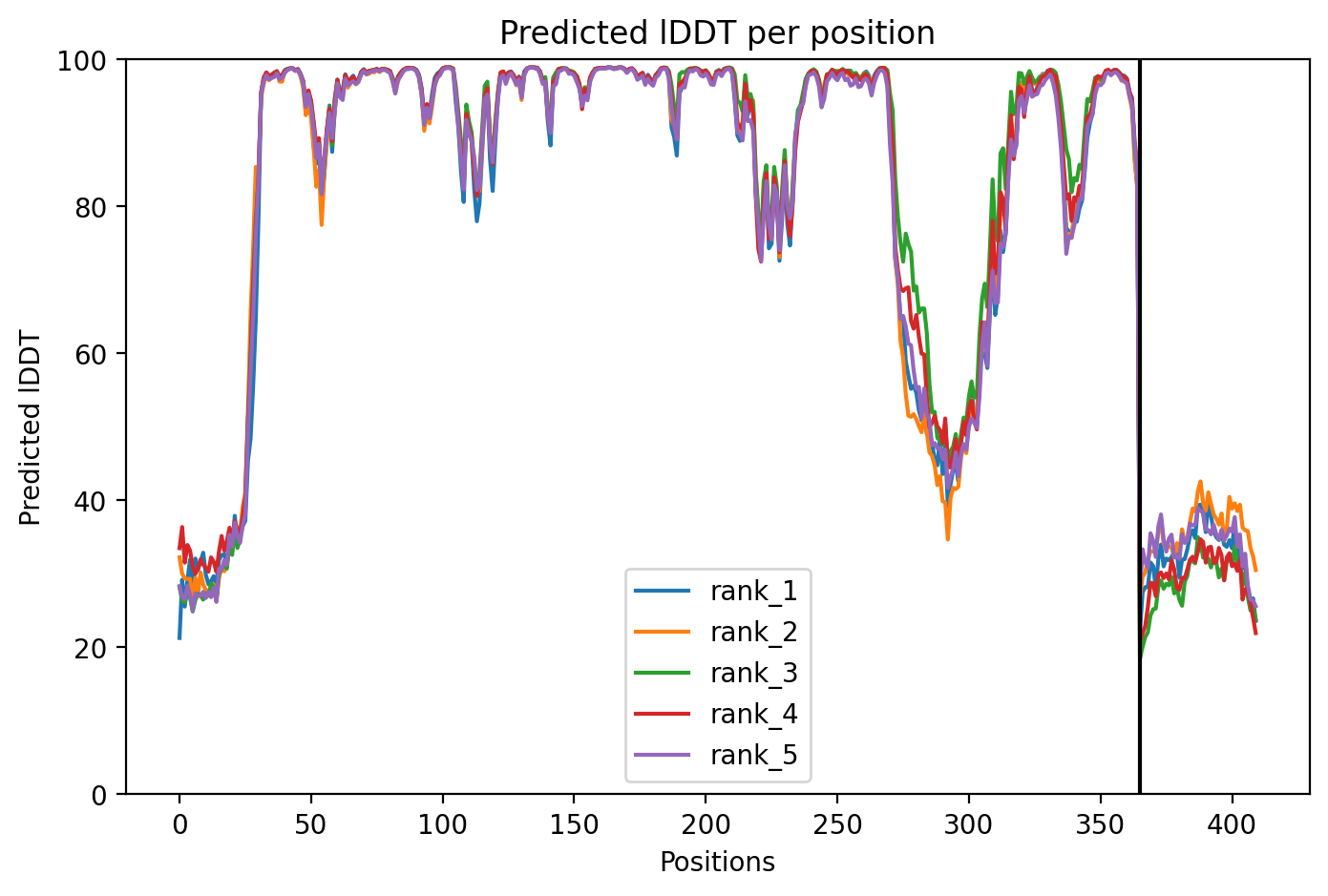

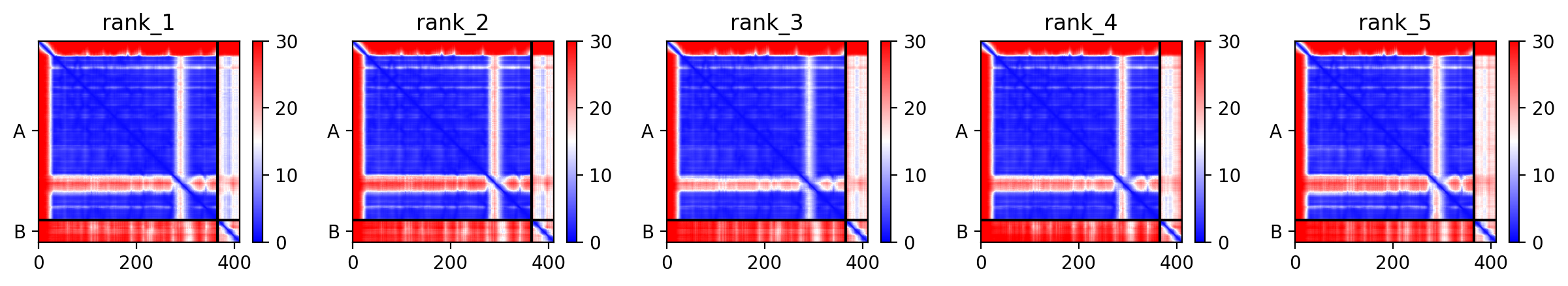


Predicted IDDT per position

Sequence coverage

A

B

**Fig. S7. MytC-PorA modelisation by Alphafold.** *A*, MytC/PorA model (color by model confidence). *B*, Statistic of AlphaFold model prediction for MytC / PorA complex.

**SI References**

20. Migliardo, F., Bourdreux, Y., Buchotte, M., Doisneau, G., Beau, J.-M., and Bayan, N. (2019) Study of the conformational behaviour of trehalose mycolates by FT-IR spectroscopy. *Chem. Phys. Lipids.***223**, 104789.

54. Van Der Peet, P. L., Gunawan, C., Torigoe, S., Yamasaki, S., and Williams, S. J. (2015) Corynomycolic acid-containing glycolipids signal through the pattern recognition receptor Mincle. *Chem. Commun*. **51**(24):5100-3.

55. Ratovelomanana-Vidal, V., Girard, C., Touati, R., Tranchier, J. P., Ben Hassine, B., and Genêt, J. P. (2003) Enantioselective Hydrogenation of β-Keto Esters using Chiral Diphosphine-Ruthenium Complexes: Optimization for Academic and Industrial Purposes and Synthetic Applications. *Adv. Synth. Catal*. 10.1002/adsc.200390021

56. Yamamoto, H., Oda, M., Nakano, M., Watanabe, N., Yabiku, K., Shibutani, M., Inoue, M., Imagawa, H., Nagahama, M., Himeno, S., Setsu, K., Sakurai, J., and Nishizawa, M. (2013) Development of vizantin, a safe immunostimulant, based on the structure-activity relationship of trehalose-6,6′-dicorynomycolate. *J. Med. Chem*. **193**(9):4507-14.

57. Brooks, D. W., Lu, L. D. ‐L, and Masamune, S. (1979) C‐Acylation under Virtually Neutral Conditions. *Angewandte Chemie International Edition in English*. 10.1002/anie.197900722

58. Seifert, T., Malo, M., Kokkola, T., Stéen, E. J. L., Meinander, K., Wallén, E. A. A., Jarho, E. M., and Luthman, K. (2020) A scaffold replacement approach towards new sirtuin 2 inhibitors. *Bioorg. Med. Chem*. **28**(2):115231.

59. Muto, S. E., and Mori, K. (2003) Synthesis of the four components of the female sex pheromone of the painted apple moth, teia anartoides. *Biosci. Biotechnol. Biochem*. **67**(7):1559-67.

60. Genêt, J. P., Pinel, C., Ratovelomanana-Vidal, V., Mallart, S., Pfister, X., De Andrade, M. C. C., and Laffitte, J. A. (1994) Novel, general synthesis of the chiral catalysts diphosphine-ruthenium (II) diallyl complexes and a new practical in situ preparation of chiral ruthenium (II) catalysts. *Tetrahedron Asymmetry*. 10.1016/0957-4166(94)80029-4

61. Radivojevic, J., Skaro, S., Senerovic, L., Vasiljevic, B., Guzik, M., Kenny, S. T., Maslak, V., Nikodinovic-Runic, J., and O’Connor, K. E. (2016) Polyhydroxyalkanoate-based 3-hydroxyoctanoic acid and its derivatives as a platform of bioactive compounds. *Appl Microbiol. Biotechnol*. **100**(1):161-72.

62. Toubiana, R., Das, B. C., Defaye, J., Mompon, B., and Toubiana, M.-J. (1975) Étude du cord-factor et de ses analogues. *Carbohydr. Res*. **44**(2):308-12.

63. Kallerup, R. S., Franzyk, H., Schiøth, M. L., Justesen, S., Martin-Bertelsen, B., Rose, F., Madsen, C. M., Christensen, D., Korsholm, K. S., Yaghmur, A., and Foged, C. (2017) Adjuvants Based on Synthetic Mycobacterial Cord Factor Analogues: Biophysical Properties of Neat Glycolipids and Nanoself-Assemblies with DDA. *Mol Pharm*. **14**(7):2294-2306.
